# Sialylation-immune-related lncRNA *LINC01605* promotes tumor-infiltrating CD8^+^ T cell exhaustion and malignancy of clear cell renal cell carcinoma

**DOI:** 10.3389/fimmu.2025.1744278

**Published:** 2026-01-16

**Authors:** Ziran Dai, Hao Zhou, Zihao Feng, Mingxiao Zhang, Zheyu Ai, Gaowei Huang, Junjie Cen, Yanping Liang, Jinhuan Wei, Wei Chen, Junhang Luo, Zhenhua Chen

**Affiliations:** 1 Department of Urology, the First Affiliated Hospital, Sun Yat-sen University, Guangdong, China; 2 Department of Urology, China-Japan Friendship Hospital, Beijing, China; 3 Department of Urology, Shenzhen People’s Hospital (the Second Clinical Medical College, Jinan University, the First Affiliated Hospital, Southern University of Science and Technology), Guangdong, China

**Keywords:** clear cell renal cell carcinoma, lncRNA, protein sialylation, tumor progression, tumor immune microenvironment

## Abstract

**Background:**

Dysregulated expression of long non-coding RNAs (lncRNAs) has been shown to play a critical role in the tumorigenicity of clear cell renal cell carcinoma (ccRCC). Meanwhile, sialylation plays a pivotal role in cancer progression and in modulating the tumor immune microenvironment. However, how sialylation-immune-related lncRNAs (SIRLs) influence tumor immune microenvironment and progression of ccRCC remains unclear.

**Methods:**

Using comprehensive cancer datasets, we identified key lncRNAs linked to both sialylation and immune modulation, constructing a prognostic risk model centered on the hub gene *LINC01605*.

**Results:**

Patients classified as high-risk showed significantly poor survival outcomes and poor response to anti-PD-1 immunotherapy compared to low-risk individuals. Functional studies established *LINC01605*’s role in enhancing tumor aggressiveness and CD8^+^ T cell exhaustion. Knockdown of *LINC01605* reduces total sialic acid levels in ccRCC cell membranes. Mechanistically, *LINC01605* recruits IGF2BP2 to increase the stability of JAK3 mRNA. Elevated JAK3 expression activates JAK3/STAT3 signaling, and phosphorylated STAT3 subsequently upregulates oncogenes (e.g., MYC) as well as sialyltransferase ST6GALNAC5—which directly increases cell membrane sialylation, a known driver of immune evasion.

**Conclusions:**

Our findings reveal the role of sialylation-immune-related lncRNAs in the immunosuppressive tumor microenvironment and cancer progression in ccRCC, providing a new framework for predicting patient outcomes and therapeutic responses.

## 1 Introduction

Renal cell carcinoma (RCC), a highly prevalent malignancy of the urinary system, represents a global public health concern. The incidence of RCC is about 5–10/100, 000 individuals, which accounts for 2–3% of all malignancies in adults. Clear cell renal cell carcinoma (ccRCC) is the most common (75%~85%) and deadly type of RCC (1, 2). Furthermore, over 25% of ccRCC patients are diagnosed to have metastasis with poor prognosis (3). Although therapies based on immune checkpoint blockade (ICB), such as PD-1 blockade, have transformed the management of advanced ccRCC, the factors that drive the development of resistance to ICB remain to be fully elucidated. Notably, in a previous study, about 80% of patients did not exhibit objective responses to PD-1 blockade therapy (4). Hence, the discovery of novel immune checkpoint targets and the development of strategies to counteract the immunosuppressive tumor microenvironment (TME) are crucial for advancing effective cancer immunotherapies.

Sialic acids are a diverse family of glycan units with similar nine-carbon backbones, typically found attached to the ends of glycan chains at cell surfaces as well as secreted by cells (5). There are currently more than 50 naturally occurring sialic acid derivatives derived from N-glycolylneuraminic acid (Neu5Gc), N-acetylneuraminic acid (Neu5Ac), and non-aminated 3-deoxy-D-glycero-D-galacto-2-nonulosonic acid (Kdn) (6, 7). Sialylation, referring to the terminal addition of sialic acid units to oligosaccharides and glycoproteins, is an important post-transcriptional modification in cellular glycosylation (8), and the levels of sialylation are tightly related to families of sialyltransferases (STs) and sialidases (NEUs) (9). Aberrantly high levels of sialic acids are expressed on the surface of tumor cells (10). Aberrant sialylation and ST activity are regarded to facilitate tumor growth, escape from apoptosis, metastasis formation and immunosuppressive TME in multiple myeloma, gynecological, and colorectal tumors (11–13). Several studies show that STs serve as prognostic biomarkers and drive tumor progression and immune evasion in ccRCC (14, 15).

Non-coding RNAs (ncRNAs), including small ncRNAs less than 200 nucleotides (nt) in length, and long non-coding RNAs (lncRNAs; more than 200 nt in length) play special roles in gene regulation but are not translated into proteins (16). Numerous studies have showed that lncRNAs play critical roles in tumors, including ccRCC (17, 18).

Although several reports show that sialylation-related lncRNAs have important functions in colorectal tumors (19, 20), few studies have examined the role of sialylation-immune-related lncRNAs (SIRLs) in the malignant progression and immunosuppressive TME of ccRCC. In this study, we obtained clinical and RNA-seq data from The Cancer Genome Atlas (TCGA), Clinical Proteomic Tumor Analysis Consortium (CPTAC) and International Cancer Genome Consortium (ICGC). Then, we utilized bioinformatic analysis methods to establish a SIRL risk model for predicting ccRCC prognosis and TME. Moreover, we investigated the biological function, and the potential underlying mechanism, of the hub gene *LINC01605* in malignant progression and immune evasion in ccRCC. Overall, the present study indicates that SIRLs are potential immune checkpoint targets and a complement for ICB treatment in ccRCC.

## 2 Materials and methods

### 2.1 Data collection and identification of sialylation-immune-related lncRNAs

The RNA-seq data, proteomic data, mutation data, and clinical information for ccRCC patient were acquired from the TCGA (https://www.cancer.gov/tcga, 528 ccRCC samples and 72 paired adjacent normal tissues), CPTAC (https://pdc.cancer.gov/pdc/, 103 ccRCC patients), and ICGC (https://icgcportal.genomics.cn/, 91 ccRCC patients) databases. We selected the TCGA, CPTAC, and ICGC databases for their comprehensive, standardized RNA-seq and clinical data on ccRCC (with TCGA-KIRC as the training cohort and CPTAC/ICGC-RECA-EU as independent validation cohorts), avoiding integrative analysis to prevent data contamination. The genes involved in the sialylation process (sialylation-related genes, SRGs) were identified from previously published articles (12, 21–23) and the Molecular Signatures Database (MSigDB) (24–26), which contains data for STs, transporters, sialic acid-binding immunoglobulin-like lectins (Siglecs), and sialidases. All gene expression data were normalized to Transcripts Per Kilobase Million (TPM) values to eliminate technical biases from sequencing depth and gene length, enabling reliable cross-sample and cross-database comparisons.

Pearson’s correlation analysis was performed between SRGs and lncRNAs based on the TCGA Kidney Renal Clear Cell Carcinoma (KIRC) cohort (528 ccRCC samples and 72 paired adjacent normal tissues). A total of 2766 sialylation-related lncRNAs were identified. The analysis of relationships between CIBERSORT (27) immune fractions and lncRNAs resulted in identification of 627 immune-related lncRNAs. Weighted correlation network analysis (WGCNA) (28) was conducted among all the lncRNAs in TCGA-KIRC cohort, and 1360 lncRNAs in modules related to phenotypes were selected. Additionally, univariate Cox proportional hazard regression analysis yielded 3357 lncRNAs. Absolute value of correlation coefficients >0.3 and P < 0.01 were used as the threshold. The intersection of the above four parts were used for further bioinformatic analysis.

### 2.2 Prognostic risk model established using sialylation-immune-related lncRNAs

To develop prognostic risk models in the TCGA-KIRC cohort, multiple machine learning methods were utilized, including “Ctree”, “Coxtime”, “Gamboost”, “Gbm”, “Glmboost”, “Glmnet”, “Loghaz”, “obliqueRSF”, “Ranger”, “Rfsrc”, “Rpart”, “Svm”, and “Xgboost” packages in R software. The LASSO-COX regression analysis in “Glmnet” had the maximum C-index, and risk scores were determined as the sum of the products of each coefficient and lncRNA expression. Tumor samples with risk scores higher than the median of all scores were categorized as the high-risk subgroup, while those with scores lower than the median value represented the low-risk subgroup. The “survival” and “survminer” R packages were utilized to perform survival analysis and plot the Kaplan-Meier survival curves. Evaluation of sensitivity and specificity of the risk model was performed by constructing receiver operating characteristic (ROC) curves and determining the area under curve (AUC). Nomograms were constructed by the “nomogramFormula” package in R software.

Multi-dimensional analyses of differences between the two risk subgroups were performed in TCGA-KIRC cohort, including with regard to their clinical characteristics, commonly mutated genes, immune infiltration estimates (ESTIMATE (29), CIBERSORT, QUANTISEQ (30) and TIMER (31)), tumor immune signatures, and tumor mutation burden (TMB). Visualization was performed using the “ggplot2”, “pheatmap”, “circlize”, “corrgram”, “maftools”, and “ggcor” packages in R software.

### 2.3 Independent validation of the sialylation-immune-related lncRNA risk model

In this study, we selected CPTAC Pan-Cancer RNA data BCM, an independent dataset containing tumor RNA-seq data for 103 ccRCC patients, of whom 93 had available clinical data; and RECA-EU, another independent dataset containing tumor RNA-seq and clinical data for 91 ccRCC patients from the ICGC. In these datasets, Kaplan-Meier survival curves and AUC were utilized to evaluate the prognostic capability, specificity, and sensitivity of the risk model, as described above.

A previous study (32) analyzed RNA-seq data of tumor samples from ccRCC patients enrolled in three clinical studies (CheckMate 009, CheckMate 010 and CheckMate 025). The dataset included 130 patients receiving mTOR inhibition (everolimus) treatment and 181 patients receiving anti-PD-1 (nivolumab) treatment. Kaplan-Meier survival curves were utilized to predict the differences in immunotherapeutic efficacy for the two risk subgroups in this dataset.

### 2.4 Gene set enrichment analysis (GSEA)

The associated signaling pathways were identified by performing GSEA in R software using “clusterprofiler” package. Gene sets were acquired from GSEA (http://www.gsea-msigdb.org/gsea/).

### 2.5 Patients and specimens

The human ccRCC tissue specimens utilized in this study were procured from Sun Yat-sen University, located in Guangzhou, China. Corresponding baseline clinical data for these patients, stratified by high and low *LINC01605* expression, are comprehensively compared in **Supplementary Table 1**.The protocol received approval from the Medical Ethics Committee of the First Affiliated Hospital, Sun Yat-sen University. ccRCC tissues were used for immunohistochemistry (IHC), quantitative real-time PCR (qRT-PCR), and flow cytometry analyses.

### 2.6 Cell lines and cell culture

The human embryonic kidney 293T cell line (293T), immortalized renal epithelial cell line (HK-2), and the human ccRCC cell lines (Caki-1, 769-P, Caki-2, 786-O, A-498 and RCCJF) were obtained from the American Type Culture Collection (ATCC). ATCC guidelines were followed for culturing all cell lines. All cell lines were analyzed by short tandem repeat (STR) profiling.

### 2.7 Biological reagents and antibodies

The detailed information on antibodies utilized for western blot, immunofluorescence, immunohistochemical staining and flow cytometry analyses was provided in **Supplementary Table 2**.

### 2.8 Lentivirus construction and cell transfection

The shRNA lentiviruses designed to target *LINC01605*, *IGF2BP2*, and *JAK3* were constructed and identified. Lentiviral packaging, infection, and puromycin selection were conducted following the same procedures as described in our previous study (18). The shRNA sequences are shown in **Supplementary Table 3**.

### 2.9 Fluorescence *in situ* hybridization (FISH) and immunofluorescence (IF) assays

Firstly, A498 and 786-O cells were fixed with 4% paraformaldehyde under ambient conditions. Subsequently, the cells were covered with an FAM-labeled *LINC01605* probe (RiboBio, China) and incubated at 37 °C overnight. After being washed three times with PBS, the cells were blocked with 5% BSA at 37 °C for 30 minutes. Then, they were incubated with an IGF2BP2 antibody at 4 °C overnight. The next day, the cells were rinsed with PBS and then incubated with the corresponding secondary fluorescent antibody at 37 °C for 1 hour. Finally, the cells were sealed with parafilm containing DAPI.

### 2.10 qRT-PCR analyses

Total RNA from patients’ tissues or cell lines were extracted with an RNA Purification Kit (EZBioscience, USA). Then, reverse transcription and qRT-PCR were performed according to the manufacturer’s instructions. GAPDH was used as an internal control. The forward and reverse primers are listed in **Supplementary Table 3**.

### 2.11 Western blot

Protein was extracted from ccRCC cell lines and western blot analysis was carried out as previously described (18).

### 2.12 Immunohistochemistry (IHC)

Tumor tissue samples were prepared by embedding them in paraffin and sectioning them to 5 mm before further processing. The antibodies mentioned above were utilized for IHC. Immunostaining images were captured as previously described (33).

### 2.13 Dual-luciferase reporter assay

A498 cells were seeded in 24-well plates and grown to approximately 80% confluence. The cells were then co-transfected with mutated *ST6GALNAC5* promoter–driven firefly luciferase reporter plasmid, a thymidine kinase promoter–driven Renilla luciferase reporter plasmid (used as an internal normalization), and either a STAT3 overexpression plasmid or an empty vector control. Transfections were performed using Lipofectamine 3000 (Invitrogen, USA) according to the manufacturer’s instructions. Forty-eight hours after transfection, luciferase activities were measured using the Dual-Luciferase Reporter Assay System (Promega, USA) following the manufacturer’s protocol.

### 2.14 RNA pull-down assay

*LINC01605* and its antisense RNAs were *in vitro* transcribed and biotin-labeled (RiboBio, China). Cell-derived protein lysates were incubated with the labeled RNAs for 1 h, followed by adding streptavidin agarose beads and another 1h incubation at room temperature. After three washes, the beads were boiled in SDS buffer, and Western blot analysis detected the captured proteins.

### 2.15 Chromatin immunoprecipitation (CHIP) assay

The chromatin immunoprecipitation (ChIP) assay was carried out using the SimpleChIP Plus Enzymatic Chromatin IP Kit (Cell signaling technology, MA, USA), strictly following the manufacturer’s instructions. In detail, after the processes of cross-linking and chromatin digestion, 10 μg of either anti-STAT3 or anti-IgG antibody was incubated with the chromatin at 4 °C overnight. Subsequently, 30 μl of protein G magnetic beads were added and the mixture was further incubated for 2 hours. Finally, the immunoprecipitated DNA was purified and analyzed by qPCR with the primers provided in **Supplementary Table 3**.

### 2.16 RNA-binding protein immunoprecipitation (RIP) assay

The RNA-binding protein immunoprecipitation (RIP) experiment was conducted with the EZMagna RIP Kit (Millipore, USA), adhering meticulously to the kit’s provided protocols. The immunoprecipitated RNA underwent qRT-PCR analysis to complete the experimental.

### 2.17 Actinomycin D Treatment

For the actinomycin D treatment, once cells reached 60% confluency in six-well plates, they were exposed to either 5 μg/mL actinomycin D or DMSO. Samples were collected at designated time intervals. Subsequently, qRT-PCR was employed to measure the mRNA expression levels.

### 2.18 Cell viability assay

Cells were seeded in 96-well plates (1×10^3^ cells/well), and cell viability at different time points (0–4 days) were measured using a CCK8 kit (HY-K030, MCE), following the manufacturer’s instruction.

### 2.19 Colony formation assay

Five-hundred untreated cells were plated in a six-well plate and incubated for 2 weeks, followed by staining with Giemsa and counting of positive colonies (>50 cells).

### 2.20 Transwell migration/invasion and wound-healing assays

Cells were plated and scratched, and photos were taken at 0 and 24 h, respectively. For migration assay, 1×10^5^ cells were plated into an 8 mm pore size Boyden chamber with serum-free medium, and 10% FBS medium was added in the bottom chamber. After 6 h, cells were fixed and stained, and the migrated cells were counted. A Boyden chamber pre-coated with Matrigel was used for invasion assay, and photos were taken after 12 h.

### 2.21 EdU assay

We utilized the EdU assay kit (Beyotime, China) to assess cell proliferation and DNA synthesis. Cell proliferation and DNA synthesis were observed using a fluorescence microscope (Olympus, Japan).

### 2.22 CD8^+^ T cell isolation and activation *in vitro*

Primary human peripheral blood mononuclear cells (PBMCs) were isolated from healthy donors (for human immune reconstitution *in vivo*) or ccRCC patients (for *in vitro* co-culture) using human lymphocyte separation medium (7111011, Dakewe), following the manufacturer’s instructions. The CD8^+^ T cell Isolation Kit (071A403.12, IPHASE) was used to purify primary human CD8^+^ T cells, following the manufacturer’s instructions. Afterward, CD8^+^ T cells were activated by adding ImmunoCult™ Human CD3/CD28/CD2 T cell activator (10970, STEMCELL) and recombinant IL-2 (C015, Novoprotein) *in vitro*.

### 2.23 Flow cytometry

CD8^+^ T cells were collected from PBMCs after being activated for three days and cultured with ccRCC cell lines for two days. After addition of Cell Activation Cocktail (423303, BioLegend) and Fc-receptor block (422301, BioLegend), the single-cell suspensions were then stained with surface markers, namely, Zombie Aqua, CD45, CD3, CD8, PD-1 and LAG-3 for 20 min, on ice. The single-cell suspensions were then treated with Fixation Buffer (420801, BioLegend) and Intracellular Staining Perm Wash Buffer (421002, BioLegend) to stain intracellular markers including TNF-α, IFN-γ, GZMB, and perforin. Samples were collected and analyzed on the BD FACS LSRFortessa Flow Cytometer, and Flowjo10 software was used to analyze the data.

### 2.24 Generation of dendritic cells (DCs) and tumor-specific CD8^+^ T cells

Monocytes were obtained from the PBMCs of HLA-A2^+^ healthy donors and cultured in VIVO medium (Lonza, USA) containing 30 ng/mL IL-4 (PeproTech, USA) and 100 ng/mL GM-CSF (PeproTech, USA). Half of the medium and cytokines were replaced every two days. DCs stimulated with 10 ng/mL TNF-α (PeproTech) were mature after 24 h. The DCs were then pulsed for another 24 h with tumor lysates from the A498 cell line by freeze-thawing with liquid nitrogen. Mature DCs were co-cultured with PBMC-isolated CD8^+^ T cells of the same donors at a ratio of 1:5 in VIVO medium containing 25 IU/mL IL-2 (PeproTech) for five days to obtain tumor-specific T cells.

### 2.25 *In vivo* mouse experiments

*In vivo* experiments in mice were approved by the Institutional Animal Care and Ethics Management Committee of Sun Yat-sen University. Four-week-old male NCG mice were used. Mice were fed under standard pathogen-free (SPF) conditions. NCG mice were subcutaneously injected with stably transfected *LINC01605*-silencing A498 cells or counterpart control A498 cells (1×10^6^). The palpable tumor volume (mm^3^) and weight was measured every three days.

For *in vivo* immune flow cytometry experiments, NCG mice were subcutaneously injected with the aforementioned A498 cells. When the tumor volume reached 100 mm³, tumor-specific CD8^+^ T cells (2.5 × 10^6^ cells per mouse) and DCs (0.5 × 10^6^ cells per mouse) were intravenously injected via the tail vein for adoptive T cell transfer, aiming to reconstitute the human immune system. When the tumor volume reached 1000 mm³ or tumor ulceration occurred, the mice were euthanized by exposure to 100% CO2 in a sealed chamber, with the flow rate of 30% of the chamber volume per minute. The tumors were then surgically excised for flow cytometry analysis.

For treatment with sialidase or the sialyltransferase inhibitor 3Fax-Neu5Ac, NCG mice were subcutaneously injected with stably transfected *LINC01605*-overexpressing A498 cells or empty vector-transfected control A498 cells (1×10^6^), followed by reconstitution of the human immune system as described above. Sialidase (10269611001, Roche) was administered via intraperitoneal injection once every three days (20 mU per mouse). 3Fax-Neu5Ac (566224, Sigma-Aldrich) was administered intraperitoneally (20 mg/kg) once daily. The mice were humanely euthanized as described above once the tumor volume reached 1000 mm³ or tumor ulceration was observed, and tumors were immediately harvested for subsequent flow cytometry analysis.

### 2.26 Single-cell sequencing data analysis

The scRNA-seq data were obtained from six ccRCC specimens described in a previous study (34). Further analysis was performed using the “Seurat” package (v 4.1) in R software. Cells with >10% of transcripts corresponding to mitochondria-encoded genes were removed. The RunUMAP function was used for low-dimensional clustering. To investigate the average gene expression within the target gene sets, we utilized the “AddModuleScore” function from the “Seurat” package. The sialylation score for each scRNA-seq sample was calculated by averaging the expression levels of SRGs. Eight inhibitory marker genes associated with CD8^+^ exhausted T cells (*PDCD1*, *LAG3*, *HAVCR2*, *TOX*, *TIGIT*, *CTLA4*, *LAYN*, and *ENTPD1*) and six marker genes indicative of CD8^+^ effector T cells (*CCL3*, *IL2RA*, *GZMB*, *TBX21*, *IFNG*, and *TNF*) were utilized to compute the exhaustion score and cytotoxicity score, respectively.

### 2.27 Statistical analysis

Bioinformatic data were analyzed and visualized using R software (v4.3.1). Experimental results were processed and plotted using GraphPad Prism v9 (GraphPad Software, La Jolla, CA, USA). Data were analyzed for normality before comparisons. For comparisons between two groups, statistical significance was determined by two-tailed paired or unpaired Student’s t-test. For multiple comparisons, one-way ANOVA followed by Tukey’s multiple comparisons or two-way ANOVA followed by Tukey’s multiple comparisons was used. Kaplan-Meier survival curves were plotted with log-rank (Mantel-Cox) test. The statistical significance of GSEA was determined by a non-parametric permutation test to calculate the nominal *P*-value for each gene set. Correlation analyses were performed using Pearson’s correlation. Differences were considered significant when p < 0.05 (∗p < 0.05, ∗∗p < 0.01 and ∗∗∗p < 0.001). Data were represented by mean ± SD. Experiments were performed on at least 3 independent samples.

## 3 Results

### 3.1 The risk prognostic signature built by sialylation-immune-related lncRNAs and validation of independent datasets

**Figure 1:**
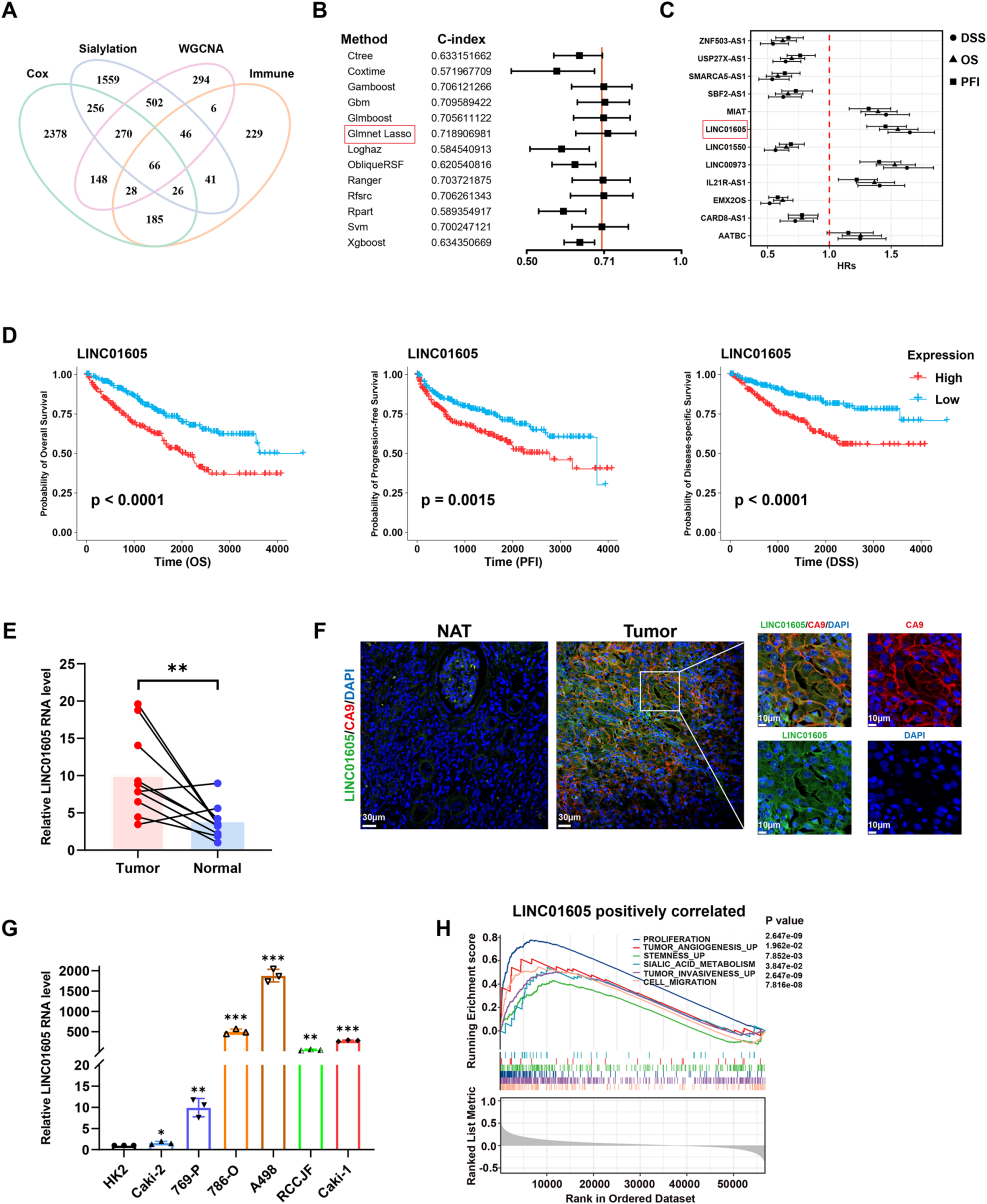
The highly expressed sialylation-immune-related lncRNA *LINC01605* in ccRCC correlates with poor prognosis. **(A)** 66 overlapping lncRNAs associated with SRGs, immune cells, phenotypes, and prognosis were identified in a Venn diagram for further investigation. **(B)** C-index of prognostic models constructed by multiple machine learning methods. **(C)** Forest plot displaying univariate Cox regression analysis results for 12 SIRLs in OS, PFI, and DSS. The HR values along with their corresponding 95% confidence intervals are shown. **(D)** Kaplan-Meier plots for overall survival (OS), progression-free interval (PFI), and disease-specific survival (DSS) were generated using the median expression of *LINC01605* as the cut-off. **(E)** Relative RNA expression of *LINC01605* in 10 pairs of human ccRCC tumors (n = 10 per group). **(F)** FISH-IF detected *LINC01605* expression in tumor and adjacent non-tumor tissues of ccRCC patients, with CA9 as the ccRCC cell marker. **(G)** Expression levels of *LINC01605* in HK-2 and different ccRCC cell lines were detected (n = 3 per group). **(H)** GSEA revealed high *LINC01605* expression to be functionally associated with enriched cell proliferation, stemness, migration, and invasion pathways. All bioinformatics analyses in **Figure 1** were based on the gene expression data (TPM values) and clinical data of 528 ccRCC samples from the TCGA-KIRC cohort. Values are presented as mean ± SD. *P*-values were calculated by log-rank (Mantel-Cox) test **(D)**, two-tailed paired Student’s t-test **(E)**, one-way ANOVA followed Tukey’s multiple comparisons **(G)**, or non-parametric permutation test **(H)**. *p < 0.05; **p < 0.01; ***p < 0.001; ns, not significant. n represents the number of biological replicates in **(E, G)**.

As **Supplementary Figure S1A** shows, there was a significant difference in the mRNA expression of 80 genes involved in sialylation between paired normal and tumor tissues from the TCGA-KIRC cohort. Principal components analysis (PCA) also revealed an obvious difference in a distinct cluster of these differentially expressed sialylation-related genes (SRGs) between the ccRCC tissues and adjacent normal tissues (**Supplementary Figures S1B, C**). Subsequently, further analyses were conducted using all samples from the TCGA-KIRC cohort. Using Pearson correlation analysis, a significant correlation was found between 2276 lncRNAs and at least one of the SRGs (**Supplementary Figure S1D**); furthermore, 627 lncRNAs were determined to be immune-related lncRNAs (**Supplementary Figure S1E**). WGCNA analysis filtered 1360 lncRNAs associated with phenotypes, such as grades and stages, in 23 co-expression modules (**Supplementary Figures S1F, G**). Further, univariate Cox regression analysis showed that 3357 lncRNAs had potential prognostic value. Sixty-six sialylation-immune-related lncRNAs (SIRLs) were filtered (**Figure 1A**), and the relationships in regard to mRNA expression between them and STs, as well as the CIBERSORT immune fractions, are shown in **Supplementary Figure S1H**.

To develop prognostic models with the TCGA-KIRC cohort, multiple machine learning methods were employed (**Figure 1B**). LASSO-COX regression analysis with the maximum C-index was used to construct a risk model on shared 66 lncRNAs. **Supplementary Figure S2A** shows the best **λ** value and 12 lncRNAs that had the highest prognostic value: *LINC01605*, *MIAT*, *AATBC*, *LINC00973*, *IL21R-AS1*, *LINC01550*, *CARD8-AS1*, *EMX2OS*, *ZNF503-AS1*, *USP27X-AS1*, *SBF2-AS1*, and *SMARCA5-AS1*. The relationship between the 12 lncRNAs and genomic location is shown in **Supplementary Figure S2B**. The relative RNA expression of the 12 lncRNAs in ccRCC cell lines was verified by qRT-PCR (**Supplementary Figure S2C**). The Kaplan-Meier plots shown in **Supplementary Figure S3A** indicate that the lncRNAs with positive coefficients in the risk model usually predict a poor prognosis. **Figure 1C** shows the forest plot of overall survival (OS), progression-free interval (PFI), and disease-specific survival (DSS). *LINC01605* was found to exhibit the highest hazard ratio (HR) values. **Supplementary Figure S3B** reveals the relationship between clinicopathological features and risk score, with a higher risk score reflecting a higher grade (p<0.0001), later stage (p<0.0001), and poorer prognosis.

The ccRCC patients in the TCGA-KIRC cohort were classified into high- or low-risk subgroups based on the median value of risk scores. **Supplementary Figure S3C** revealed that the high-risk subgroup exhibited a poorer OS (p < 0.0001), PFI (p < 0.0001) and DSS (p < 0.0001) and also showed the AUC of OS, PFI, and DSS at 1, 3, and 5 years. As shown in the ROC curves, OS, PFI, and DSS had a high overall AUC around 0.8, 0.7, and 0.75, respectively. Validation of the risk model was performed by applying it to two independent datasets (CPTAC and ICGC) (**Supplementary Figure S3D**) (data on PFI and DSS were not collected). Similar to the TCGA dataset, the Kaplan-Meier curve revealed that the high-risk subgroup had a worse OS (p=0.033 and p=0.037, respectively). Moreover, this risk model also exhibited a similar AUC to that of the TCGA dataset, indicating that the model can also be applied to other datasets.

### 3.2 The risk prognostic model correlates with immune landscapes and response to immunotherapy in ccRCC

Analysis of TMB based on TCGA-KIRC somatic mutation data showed that some commonly mutated ccRCC genes exhibited significant differences between the two risk subgroups. The high-risk subgroup had a 13% and 8% higher mutation rate of BAP1 and SETD2, respectively, than the low-risk subgroup (**Supplementary Figure S4A**). Interestingly, there was no significant difference (p=0.086) in TMB value between the two risk subgroups (**Supplementary Figure S4A**), suggesting that in ccRCC, the TMB may not have correlate strongly with prognosis. A previous research has also shown that TMB cannot predict survival well after immunotherapy in ccRCC (35).

Both ESTIMATE tumor purity score and ESTIMATE immune score in the high-risk subgroup were higher than those in the low-risk subgroup (p<0.001), which indicates that the high-risk subgroup had more immune infiltration (**Supplementary Figure S4B**). Using the TIMER and QUANTISEQ algorithm, we observed greater infiltration of CD8^+^ T cells and less infiltration of CD4^+^ T cells and B cells in high-risk tumors than in low-risk tumors (**Supplementary Figure S4C**). Previous studies showed a close correlation of high CD8^+^ T cell infiltration in ccRCC with poor prognosis (36, 37). As **Supplementary Figure S4D** shows, the high-risk subgroup had higher expression of CD8^+^ T cell exhaustion markers, especially *PDCD1*, *LAG3*, and *TIGIT*. The above results reveal that the risk score was positively correlated with the immunosuppressive microenvironment. We next applied the model in an independent dataset CheckMate. As **Supplementary Figure S4E** shows, the low-risk group treated with nivolumab had the most favorable prognosis and highest proportion of patients who experienced clinical benefit. In general, our risk model has a meaningful prognostic value for immunotherapy. (Detailed descriptions of the data used in the analyses in this section are provided in the legend of **Supplementary Figure S4** and the Methods section).

### 3.3 *LINC01605*, hub gene of the risk model, is associated with malignant progression of ccRCC

**Figure 2:**
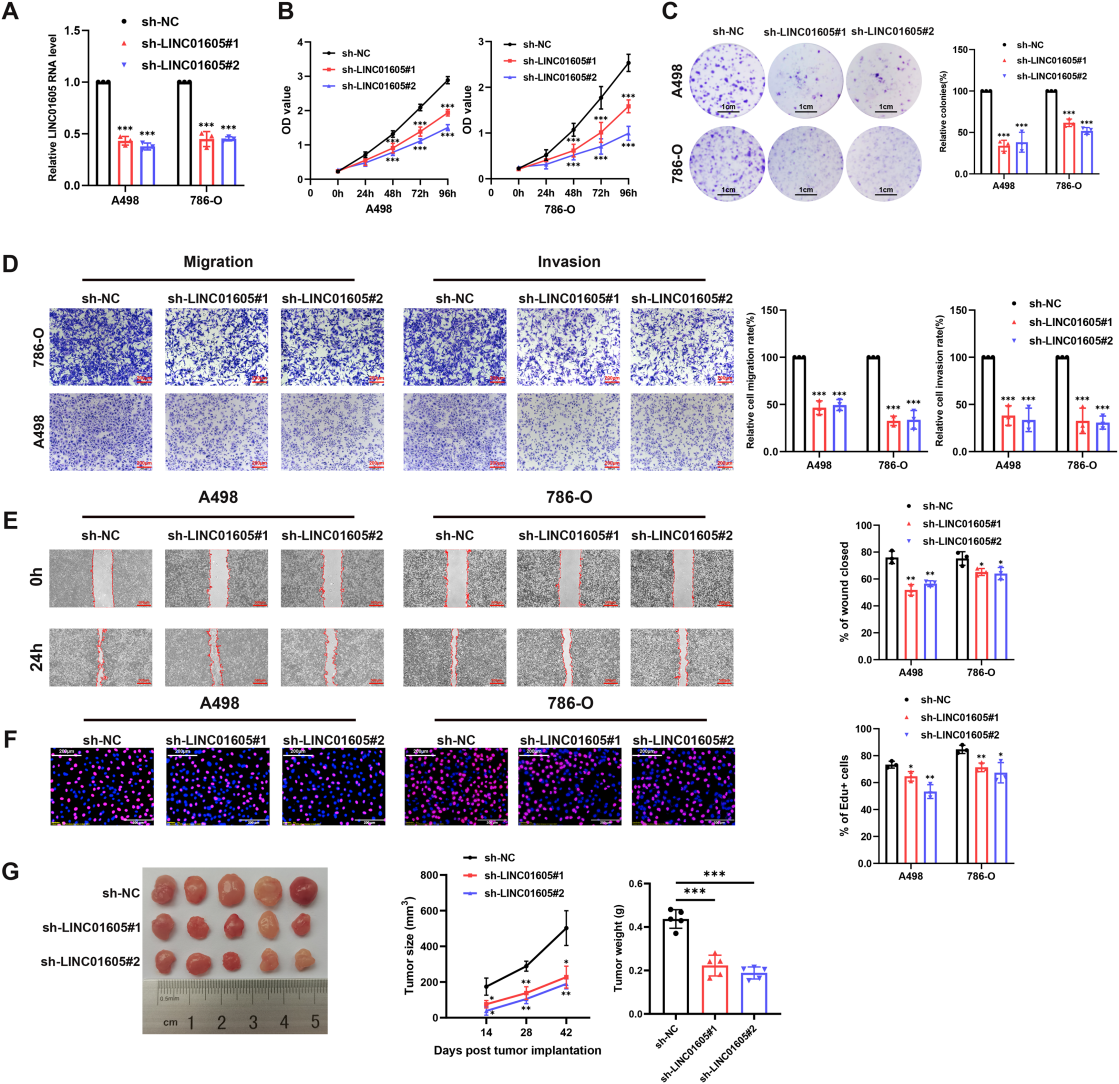
*LINC01605* is associated with the malignant progression of ccRCC cells. **(A)***LINC01605* was silenced in A498 and 786-O cell lines by two different shRNAs (n = 3 per group). **(B, C)** CCK8 and colony formation assays were performed in *LINC01605*-knockdown and counterpart control groups (n = 3 per group). **(D, E)** Transwell migration/invasion and wound-healing assays showed that knockdown of *LINC01605* impaired mobility and invasiveness of ccRCC cell lines (n = 3 per group). **(F)** EdU assay showed that cell proliferation and DNA synthesis decreased in *LINC01605*-silenced ccRCC cell lines (n = 3 per group). **(G)** Knockdown of *LINC01605* in A498 cells inhibited the growth of A498-derived xenograft *in vivo* (n = 5 per group). Tumor growth curves, tumor weights and statistical analysis are shown. Values are presented as mean ± SD. *P*-values were determined by one-way ANOVA followed Tukey’s multiple comparisons [**(A-G)** one comparison per time point for **(B**, **G)**]. *p < 0.05; **p < 0.01; ***p < 0.001; ns, not significant. n represents the number of biological replicates in **(A-G)**.

*LINC01605* was found to have the largest prognostic hazard ratio (**Figure 1C**), and was used for subsequent experimental verification. Similar to those with high risk scores, patients with high expression of *LINC01605* also exhibited a poorer prognosis in the TCGA-KIRC cohort (**Figure 1D**). The heightened expression of *LINC06105* was confirmed in paired ccRCC tissue samples via qRT-PCR validation (**Figure 1E**). Meanwhile, FISH-IF analysis showed higher *LINC01605* expression in tumor tissues than in adjacent non-tumor tissues of ccRCC patients, with CA9 as the ccRCC cell marker (**Figure 1F**). To identify the most appropriate ccRCC cell lines, we assessed the expression of *LINC01605* across a panel of ccRCC cell lines (**Figure 1G**). GSEA analysis in TCGA-KIRC showed that high *LINC01605* expression was linked to the enrichment of cell proliferation, stemness, migration, and invasion pathways (**Figure 1H**). To validate these oncogenic functions of *LINC01605*, we conducted loss-of-function experiments. *LINC01605* silencing in A498 and 786-O cells (**Figure 2A**) dramatically suppressed cell proliferation and colony formation, respectively (**Figures 2B, C**). We also conducted transwell migration and Matrigel invasion assays and found that *LINC01605* knockdown impaired the mobility of A498 and 786-O cells (**Figure 2D**). In addition, wound-healing assays validated that *LINC01605* knockdown inhibited migration and invasion of A498 and 786-O cells (**Figure 2E**). As shown in **Figure 2F**, EdU assay results revealed that cell proliferation and DNA synthesis decreased in *LINC01605*-silenced cell lines. The function of *LINC01605* was verified *in vivo*: knockdown of *LINC01605* in A498 cells dramatically inhibited the growth rate of A498-derived xenografts in NCG mice (**Figure 2G**). Overall, our findings suggest that *LINC01605* plays a significant role in the malignant progression of ccRCC, both *in vitro* and *in vivo*.

### 3.4 *LINC01605* induces CD8^+^ T cell exhaustion and immunosuppressive TME in ccRCC

**Figure 3:**
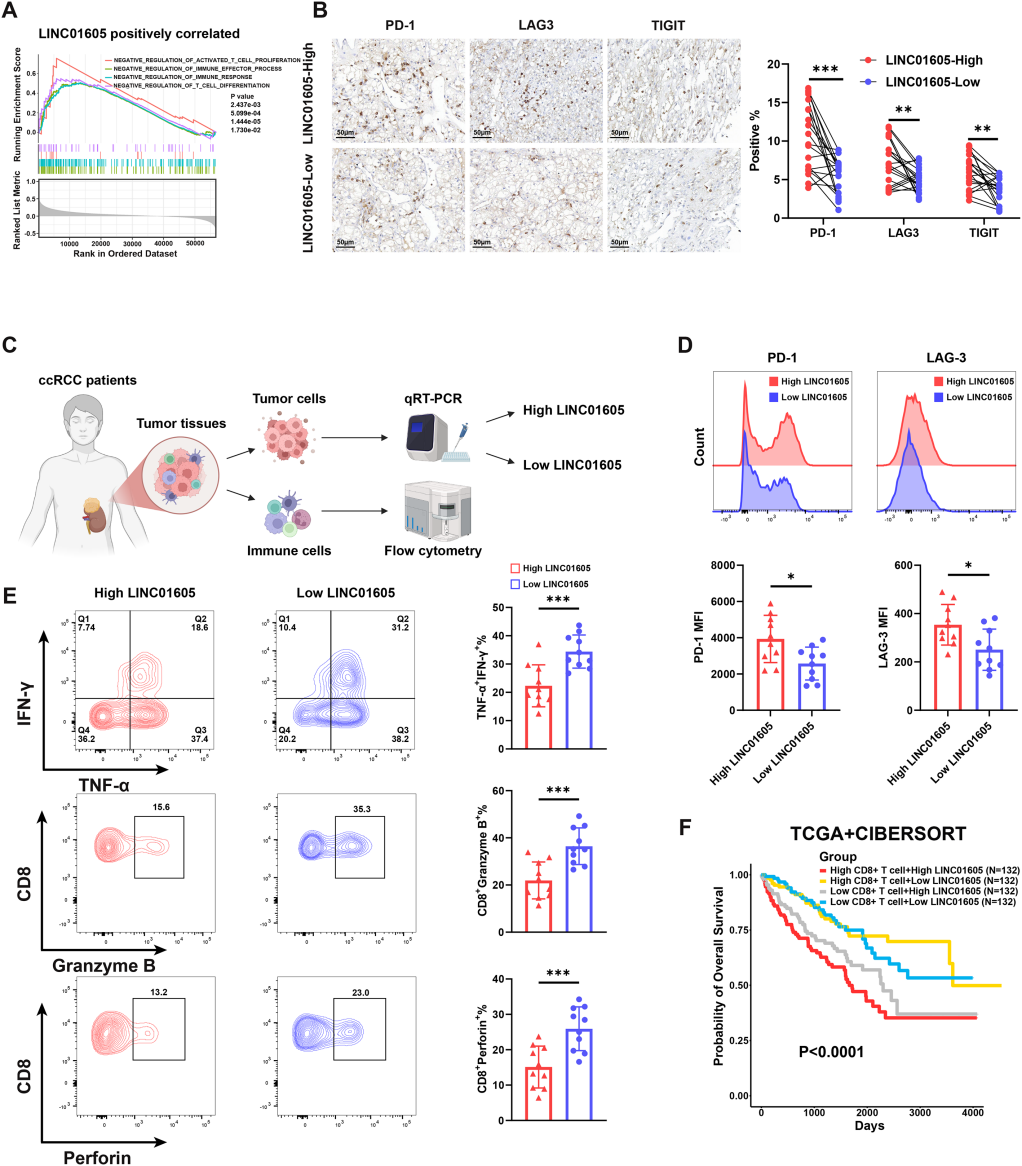
*LINC01605* correlates with CD8^+^ T cell exhaustion and immunosuppressive TME in ccRCC. **(A)** GSEA analysis in the TCGA-KIRC cohort (n=528) showed that high expression of *LINC01605* is involved in pathways associated with negative regulation of the immune response and inhibition of T cell activation. **(B)** Immunohistochemistry was used to detect the correlation between *LINC01605* and tumor-infiltrating CD8^+^ T cell exhaustion markers (PD-1, LAG3, and TIGIT) in 40 clinical specimens of ccRCC (n = 20 per group). **(C)** Schematic diagram of flow cytometry analysis of tumor samples from ccRCC patients classified into *LINC01605* high-expression and low-expression groups. **(D)** Flow cytometry results showed that infiltrating CD8^+^ T cells in tumor samples with high *LINC01605* expression had higher expression levels of PD-1 and LAG-3 (n = 10 per group). **(E)** Flow cytometry results demonstrated that infiltrating CD8^+^ T cells in tumor samples with high *LINC01605* expression exhibited lower expression levels of TNF-α, IFN-γ, GZMB, and perforin (n = 10 per group). **(F)** Kaplan–Meier analysis of four groups of patients from the TCGA-KIRC cohort (n=528) with different CIBERSORT scores of CD8^+^ T cell and expression of *LINC01605*. Values are presented as mean ± SD. *P*-values were calculated by non-parametric permutation test **(A)**, two-tailed unpaired Student’s t-test **(B, D, E)**, or log-rank (Mantel-Cox) test **(F)**. *p < 0.05; **p < 0.01; ***p < 0.001; ns, not significant. n represents the number of samples in **(A, F)** and the number of biological replicates in **(B, D, E)**.

Tumor-infiltrating CD8^+^ T cells in ccRCC are mostly exhausted and unable to normally exercise their function of killing tumor cells, which leads to immune escape. GSEA analysis results from TCGA-KIRC cohort, shown in **Figure 3A**, reveal that high expression of *LINC01605* is involved in pathways associated with inhibition of T cell activation. We further discovered whether there is crosstalk between *LINC01605* with CD8^+^ T cells. The correlation between *LINC01605* and PD-1, LAG3, and TIGIT expression on tumor-infiltrating CD8^+^ T cells in 40 ccRCC samples was examined by IHC. Samples were categorized into *LINC01605* high-expression and low-expression groups with the median value as the cutoff. Results showed that the expression levels of CD8^+^ T cell exhaustion indicators in tumors with high *LINC01605* expression were significantly elevated (**Figure 3B**). Subsequently, we performed flow cytometry analysis on tumor tissues with high and low *LINC01605* expression from ccRCC patients (**Figure 3C**). CD3^+^/CD8^+^ cells were characterized as CD8^+^ T cells (**Supplementary Figure S5A**). Results showed that CD8^+^ T cells infiltrating *LINC01605*-high-expression tumors had higher PD-1 and LAG-3 expression, and lower levels of TNF-α, IFN-γ, GZMB, and perforin (**Figures 3D, E**). In the CheckMate cohorts, patients treated with nivolumab who showed low *LINC01605* expression had the best prognosis (p=0.023) (**Supplementary Figure S5B**). In addition, we combined the TCGA-KIRC cohort with CIBERSORT results and divided all patients into four groups based on CD8^+^ T cell infiltration scores and *LINC01605* expression levels. Notably, although high CD8^+^ T cell infiltration typically indicates a poor prognosis in ccRCC (36, 38), patients belonging to the CD8^+^-high and *LINC01605*-low groups demonstrated the most favorable prognosis (p < 0.0001) (**Figure 3F**).

**Figure 4:**
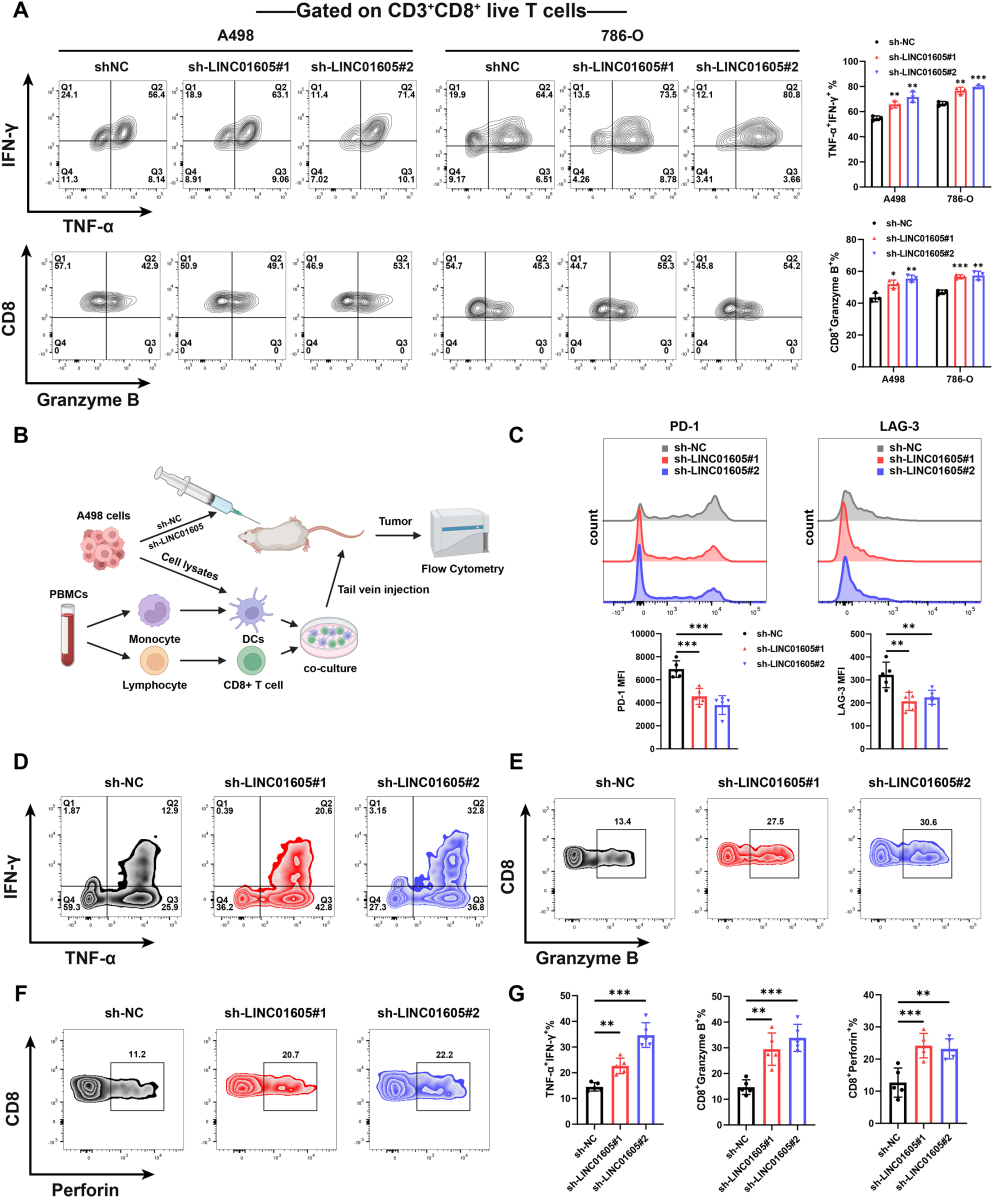
*LINC01605* regulated CD8^+^ T cell exhaustion and function *in vitro* and *in vivo*. **(A)** Flow cytometry results showed that *LINC01605* knockdown in A498 and 786-O cell lines promoted the secretion of IFN-γ, TNF-α, and GZMB by human CD8^+^ T cells *in vitro* (n = 3 per group). **(B)** Schematic diagram of PBMC-humanized NCG mouse construction. **(C)** Flow cytometry results revealed that infiltrating CD8^+^ T cells in tumor samples with *LINC01605* knockdown exhibited lower expression of PD-1 and LAG-3 (n = 5 per group). **(D-G)** Flow cytometry results demonstrated that infiltrating CD8^+^ T cells in tumor samples with *LINC01605* knockdown displayed increased expression of TNF-α, IFN-γ, GZMB, and perforin (n = 5 per group). Values are presented as mean ± SD. *P*-values were determined by one-way ANOVA followed Tukey’s multiple comparisons **(A, C, G)**. *p < 0.05; **p < 0.01; ***p < 0.001; ns, not significant. n represents the number of biological replicates in **(A**, **C-G)**.

We further investigated whether *LINC01605* could regulate the exhaustion status and function of CD8^+^ T cells both *in vitro* and *in vivo*. PBMCs were extracted from ccRCC patients to isolate CD8^+^ T cells, which were then cultured with A498 and 786-O cell lines. CD8^+^ T cells co-cultured with *LINC01605*-silenced A498 and 786-O cells exhibited upregulated expression of TNF-α, IFN-γ, GZMB, and perforin (**Figure 4A**, **Supplementary Figure S5C**). *In vivo*, we utilized PBMC-humanized NCG mice subcutaneously inoculated with A498 cells (with manipulated *LINC01605* expression) to evaluate the regulatory role of *LINC01605* on CD8^+^ T cells (**Figure 4B**). Flow cytometry analysis of the resulting tumors showed that knockdown of *LINC01605* reduced the expression levels of PD-1 and LAG-3 in infiltrating CD8^+^ T cells, while increasing those of TNF-α, IFN-γ, GZMB, and perforin (**Figures 4C-G**). Taken together, the results reveal that *LINC01605* overexpression in ccRCC induces CD8^+^ T cell exhaustion, highlighting the potential value of *LINC01605* as a therapeutic target for improving the efficacy of immunotherapy.

### 3.5 Sialylation is associated with CD8^+^ T cell exhaustion in ccRCC, and *LINC01605* silencing decreases sialic acid levels on the cell membrane

**Figure 5:**
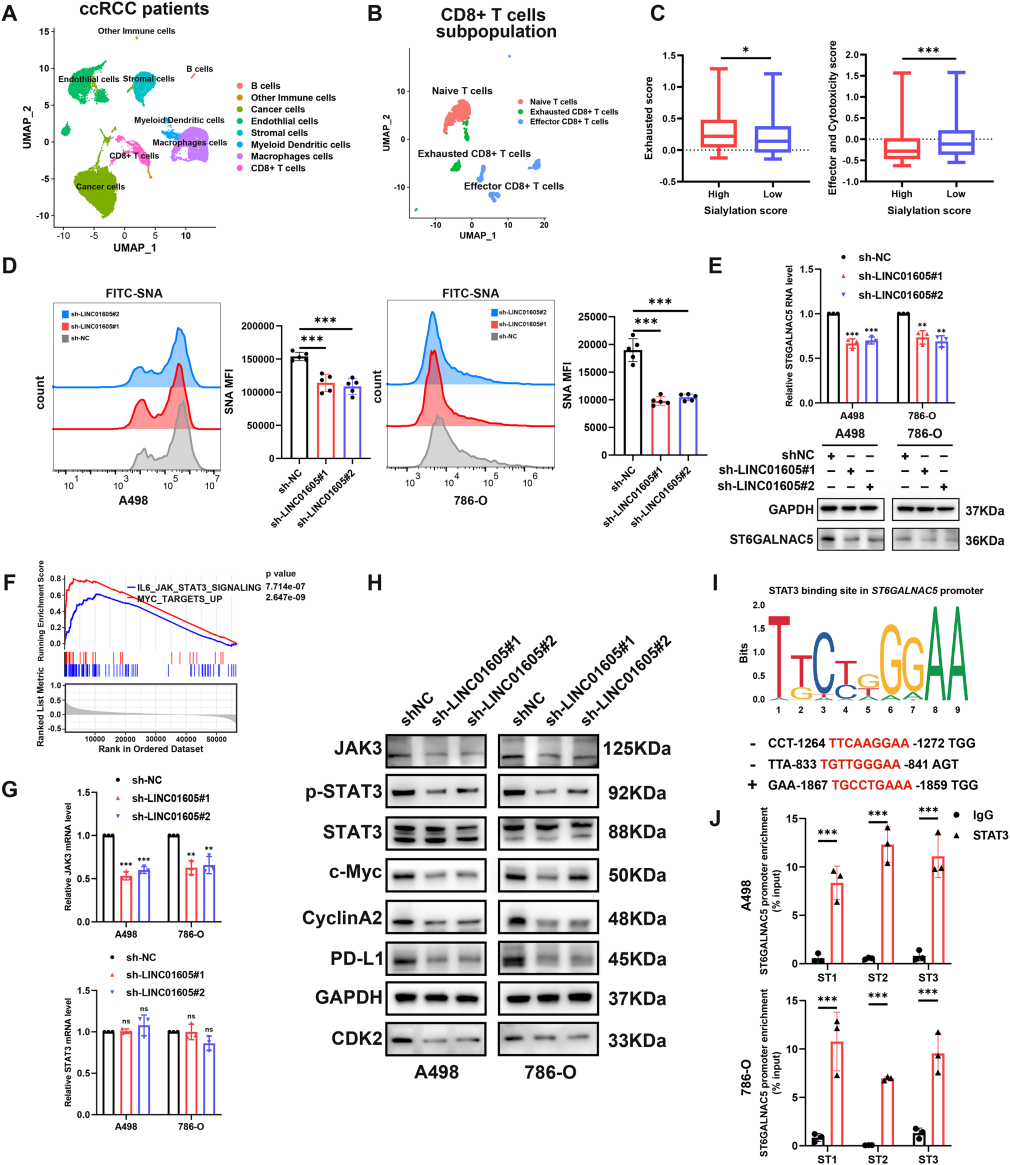
*LINC01605* is associated with sialic acid levels and involved in JAK3/STAT3 signaling. **(A, B)** Uniform manifold approximation and projection (UMAP) plots of ccRCC samples and tumor-infiltrating CD8^+^ T cell subpopulation. **(C)** ccRCC samples with highly sialylated tumor cells had higher exhaustion scores and lower effector and cytotoxicity scores in the corresponding CD8^+^ T cell subpopulation. **(D)** Flow cytometry results indicated that *LINC01605* silencing decreased sialic acid levels on the cell membrane of A498 and 786-O cell lines (n = 5 per group). **(E)** qRT-PCR and western blotting showed that *LINC01605* expression was correlated with mRNA and protein expression of ST6GALNAC5 (n = 3 per group). **(F)** GSEA analysis in the TCGA-KIRC cohort (n=528) revealed that high *LINC01605* expression was related to the IL6/JAK/STAT3 and MYC target pathways. **(G)** The mRNA expression of *JAK3* and *STAT3* was detected using qRT-PCR in *LINC01605*-silenced ccRCC cell lines (n = 3 per group). **(H)** Western blotting was performed to detect the expression of JAK3, STAT3, phosphorylated STAT3, and downstream genes of the pathway after *LINC01605* knockdown in ccRCC cell lines. **(I)** Binding sites of STAT3 at the *ST6GALNAC5* promoter were predicted by JASPAR. **(J)** ChIP assays demonstrated that STAT3 bound to the *ST6GALNAC5* promoter in A498 and 786-O cells (n = 3 per group). Values are presented as mean ± SD. *P*-values were calculated by two-tailed unpaired Student’s t-test **(C, J)**, one-way ANOVA followed Tukey’s multiple comparisons **(D, E, G)**, or non-parametric permutation test **(F)**. *p < 0.05; **p < 0.01; ***p < 0.001; ns, not significant. n represents the number of samples in **(F)** and the number of biological replicates in **(D-E, G, J)**.

Recent studies have demonstrated that sialylation of tumor cells can influence immune cell function, thereby contributing to an immunosuppressive TME (39). To explore whether sialylation affects immune cells, especially T cells, in ccRCC, we analyzed six ccRCC specimens from a scRNA-seq dataset (34). Cell clustering analysis revealed eight subpopulations of tumor samples and three subpopulations of CD8^+^ T cells (**Figures 5A, B**). All specimens were divided into high- and low-sialylation groups based on median value of sialylation score (**Supplementary Figures S6A, B**). Visualization of the exhaustion and cytotoxicity scores validated diminished dysfunction and elevated effector function of CD8^+^ T cells in low-sialylation tumor samples (**Supplementary Figure S6C**, **Figure 5C**). As shown in **Figure 1H**, *LINC01605* expression was also related to sialylation. We utilized flow cytometry to investigate the sialylation patterns on various cell surfaces. Cell surface staining with FITC-SNA—a specific lectin that binds to α-2, 6 linked sialic acid—revealed that *LINC01605*-silenced A498 and 786-O cell lines had significantly lower mean fluorescence intensities (MFI) than the control group (**Figure 5D**). These results show that *LINC01605* inhibition decreased sialic acid levels on the cell membranes of ccRCC cells. *ST6GALNAC5* was one of the STs most strongly related to *LINC01605* based on the TCGA-KIRC cohort and the CPTAC cohort (**Supplementary Figure S6D**), and we discovered that silencing *LINC01605* resulted in the downregulation of *ST6GALNAC5* in A498 and 786-O cell lines (**Figure 5E**). Furthermore, to investigate whether *LINC01605*-mediated regulation of cell surface sialic acid levels in ccRCC cells could account for its effects on the exhaustion and function of tumor-infiltrating CD8^+^ T cells, we supplemented sialidase or the sialyltransferase inhibitor 3Fax-Neu5Ac treatment in our aforementioned *in vivo* PBMC-humanized NCG mouse model. Flow cytometry analysis of tumors harvested from these mice demonstrated that sialidase or 3Fax-Neu5Ac treatment reversed the *LINC01605* overexpression-induced increase in PD-1 and LAG-3 expression levels, as well as the decrease in TNF-α, IFN-γ, GZMB, and perforin expression, in tumor-infiltrating CD8^+^ T cells (**Supplementary Figures S7A-F**). Taken together, we clarify that increasing sialic acid levels is one of the pathways by which *LINC01605* mediates CD8^+^ T cell exhaustion.

### 3.6 *LINC01605* is associated with the JAK3/STAT3 signaling pathway.

We next explored the molecular mechanism underlying the oncogenic role of *LINC01605* by performing GSEA on the RNA-seq data of the KIRC cohort in the TCGA database. It was found that *LINC01605* levels were significantly positively correlated with IL6-JAK-STAT3 and MYC target pathways (**Figure 5F**). Among the four different JAK molecules, JAK3 has the strongest correlation with *LINC01605* expression based on the CPTAC cohort (**Supplementary Figure S8A**). Furthermore, qRT-PCR and western blotting were performed to detect the expression of JAK3 and STAT3 after *LINC01605* knockdown in A498 and 786-O cell lines. The results showed that mRNA and protein expression levels of JAK3 were significantly decreased; however, there was no significant change in STAT3 (**Figure 5G**). Western blotting results also revealed that the expression levels of phosphorylated STAT3 and well-known STAT3 downstream targets, such as c-Myc, Cyclin A2, CDK2, and PD-L1 (40, 41), were significantly decreased after *LINC01605* knockdown (**Figure 5H**). To further investigate whether *ST6GALNAC5* is a downstream target gene of the JAK3/STAT3 pathway, we analyzed the *ST6GALNAC5* promoter sequence using JASPAR and identified three STAT3 binding sites (within -1264 to -1272 bp, -833 to -841 bp, and -1867 to -1859 bp upstream of the *ST6GALNAC5* transcription start site) (**Figure 5I**). Chromatin immunoprecipitation followed by qPCR (ChIP-qPCR) further confirmed STAT3-specific binding to the *ST6GALNAC5* promoter region in A498 and 786-O cells (**Figure 5J**). Subsequently, Dual-luciferase reporter gene assay revealed that the overexpression of STAT3 significantly enhanced the luciferase activity of the binding site 1 reporter construct (-1264 to -1272 bp upstream of the *ST6GALNAC5* transcription start site) (**Supplementary Figure S8B**). These results indicate that *LINC01605* can participate in the JAK3/STAT3 pathway by regulating JAK3 expression, and that *ST6GALNAC5* is a downstream target of STAT3.

### 3.7 *LINC01605* upregulates JAK3 expression by recruiting IGF2BP2 to increase the stability of JAK3 mRNA

**Figure 6:**
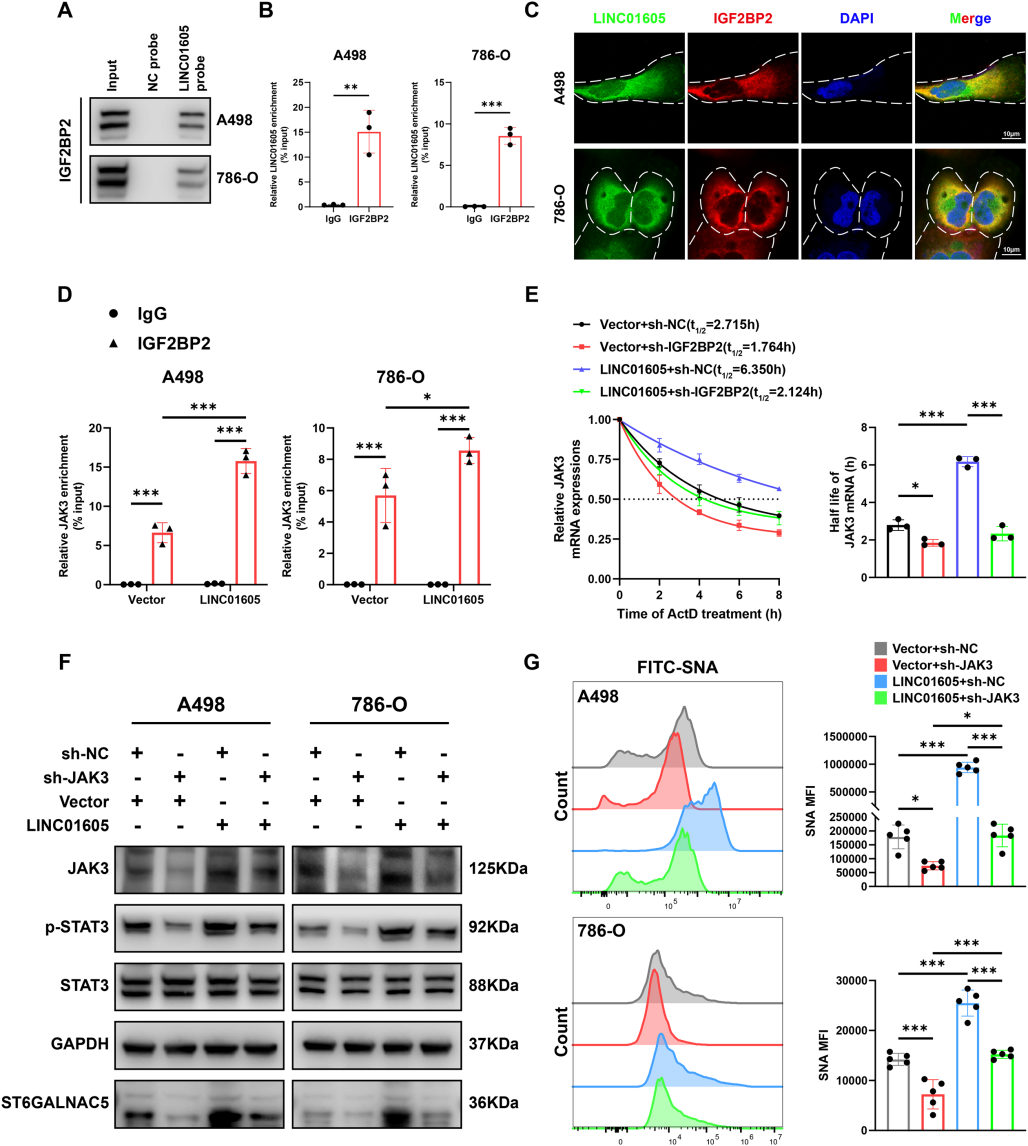
*LINC01605* promotes the stability of JAK3 mRNA by recruiting IGF2BP2 and regulates the level of cell membrane sialylation via the JAK3/STAT3 pathway. **(A)** The interaction between *LINC01605* and IGF2BP2 in A498 and 786-O cells was detected by RNA pull-down assay followed by western blot. **(B)** RNA immunoprecipitation (RIP) and qRT-PCR assays showed specific binding between IGF2BP2 and *LINC01605* (n = 3 per group). **(C)** Representative FISH-IF images revealed the cytoplasmic colocalization of *LINC01605* and IGF2BP2 in A498 and 786-O cells. **(D)** RIP assays demonstrated that overexpression of *LINC01605* affects the interaction between IGF2BP2 and JAK3 mRNA (n = 3 per group). **(E)***LINC01605* overexpression markedly increased JAK3 mRNA stability in A498 cells, with this effect abrogated by sh-IGF2BP2 (n = 3 per group). **(F)** Western blot results demonstrated that knockdown of JAK3 largely attenuated the upregulating effect of *LINC01605* overexpression on ST6GALNAC5. **(G)** SNA staining results revealed that JAK3 knockdown largely diminished the promoting effect of *LINC01605* overexpression on the sialylation level of cell membranes in ccRCC cells (n = 5 per group). Values are presented as mean ± SD. *P*-values were calculated by two-tailed unpaired Student’s t-test **(B)**, two-way ANOVA followed Tukey’s multiple comparisons **(D)**, or one-way ANOVA followed Tukey’s multiple comparisons **(E, G)**. *p < 0.05; **p < 0.01; ***p < 0.001; ns, not significant. n represents the number of biological replicates in **(B, D, E, G)**.

To explore the underlying mechanisms by which *LINC01605* modulates JAK3 expression levels, we first characterized its subcellular localization and identified its predominant distribution in the cytoplasm (**Figure 1F**). Cytoplasmic lncRNAs have been shown to generally recruit RNA-binding proteins (RBPs) to enhance mRNA stability and upregulate their expression (42, 43). Therefore, we hypothesize that *LINC01605* can recruit RBPs to stabilize JAK3 mRNA. Using the starBase website, we screened out three RBPs that bind to *LINC01605* (IGF2BP2, YTHDC1, and RBFOX2) with criteria of pan-Cancer ≥15 and CLIP-Data ≥3. Among them, IGF2BP2 is predominantly localized in the cytoplasm and has been widely reported to bind and stabilize mRNAs (44, 45). Next, we used RNA pull-down and RIP assays to demonstrate the interaction between IGF2BP2 and *LINC01605* in A498 and 786-O cells (**Figures 6A, B**). Furthermore, FISH-IF assay confirmed the colocalization of *LINC01605* and IGF2BP2 in the cytoplasm of A498 and 786-O cells (**Figure 6C**). Meanwhile, RIP assay results demonstrated that IGF2BP2 binds to JAK3 mRNA in A498 and 786-O cells, and overexpression of *LINC01605* enhances this binding (**Figure 6D**). Actinomycin D assay results indicated that overexpression of *LINC01605* significantly enhanced JAK3 mRNA stability, an effect that was dependent on IGF2BP2 (**Figure 6E**). Notably, sequence BLAST analysis identified complementary sequences suggesting that *LINC01605* may bind to JAK3 mRNA, and this interaction was subsequently confirmed by RNA pull-down assay (**Supplementary Figures S9A, B**). Therefore, we speculated that *LINC01605* may function as a scaffold molecule to facilitate the recruitment of IGF2BP2 onto JAK3 mRNA, thereby enhancing the stability of JAK3 mRNA. Collectively, these results reveal that *LINC01605* facilitates the recruitment of IGF2BP2 to stabilize JAK3 mRNA.

### 3.8 *LINC01605* regulates cell membrane sialylation via the JAK3/STAT3 pathway

To investigate whether the regulatory effect of *LINC01605* on sialylation in ccRCC cells is mediated through the JAK3/STAT3 pathway, we knocked down JAK3 in A498 and 786-O cells with *LINC01605* overexpression. The results of western blotting and SNA staining revealed that JAK3 knockdown attenuated the upregulation of ST6GALNAC5 expression and the increase in cell membrane α-2, 6 sialylation levels mediated by *LINC01605* overexpression (**Figures 6F, G**). In summary, these results suggest that the high expression of *LINC01605* in ccRCC cells promotes the level of cell membrane sialylation by upregulating the expression of JAK3.

## 4 Discussion

Sialylation refers to the covalent addition of sialic acids to the ends of glycoproteins. It is a biologically important modification that is involved in embryonic development, neural development, reprogramming, tumorigenesis, and the immune response (39, 46). Aberrant sialylation is one of the universal features of cancer and plays an important biological role in tumor transformation, growth, metastasis, immune evasion and drug resistance (11, 12, 22, 47). Sialylation has often been studied in tumors such as colon cancer and breast cancer, but is rarely examined in ccRCC. However, a previous study showed that recognition of sialic acid on the cell surface offers a potential approach to bedside rapid detection of RCC in clinical applications (48). This finding suggests that sialylation may also have applications in the prediction and clinical treatment of ccRCC.

Numerous non-coding RNAs, such as lncRNAs, circRNAs, and piRNAs, exert critical functions in renal cell carcinoma tumorigenesis (49–51). A close correlation has been identified between lncRNAs and sialylation in tumors (19, 20), and lncRNAs may also affect sialylation in ccRCC. Accordingly, lncRNAs related to sialylation and immune cells might serve as critical therapeutic targets in cancer treatment, making it important to explore their potential utility in this context. Herein, based on the TCGA-KIRC dataset, we identified 12 SIRLs and constructed a risk model. Hub gene *LINC01605* of the model can promote CD8^+^ T cell exhaustion and malignant progression of ccRCC. Other lncRNAs of the model, such as *MIAT*, may also be involved in the immune escape process, according to a previous study (52).

Previous studies have demonstrated that *LINC01605* exerts oncogenic effects in multiple tumor types, including pancreatic ductal adenocarcinoma (PDAC), cervical cancer, triple-negative breast cancer (TNBC), and colorectal cancer (CRC). However, it also exhibits tumor-suppressive properties in certain malignancies, such as esophageal cancer, indicating a context-dependent role of *LINC01605* in tumorigenesis. The oncogenic mechanisms of *LINC01605* have been partially elucidated in different tumors: in PDAC, *LINC01605* promotes cancer cell proliferation and migration by activating the mTOR signaling pathway (53); in cervical cancer, it enhances malignant phenotypes (e.g., proliferation and invasion) through the miR-149-3p/WNT7B ceRNA axis (54); in TNBC, it facilitates aerobic glycolysis via lactate dehydrogenase A (LDHA), thereby augmenting cancer cell proliferation, migration, and invasion (55); and in CRC, overexpression of *LINC01605* interacts with the METTL3 protein to induce m^6^A modification of SPTBN2 mRNA, which in turn enhances cancer cell proliferative and metastatic capacities (56). In contrast, in esophageal squamous cell carcinoma (ESCC), *LINC01605* inhibits tumorigenesis by regulating the differentiation, proliferation, and migration of squamous cells (57). Notably, to the best of our knowledge, no prior studies have systematically investigated the regulatory crosstalk between *LINC01605* and sialylation, nor have they explored its specific role in modulating TME remodeling in ccRCC. Compared with other well-characterized lncRNAs in ccRCC, *LINC01605* features a distinct sialylation-centered regulatory mode. For instance, MALAT1 promotes tumor progression mainly as a ceRNA by targeting the miR-203/BIRC5 axis (58), while *LINC01138* interacts with PRMT5 to drive lipid desaturation and correlates with immune cell infiltration (59). In contrast, our study is the first to reveal that *LINC01605* uniquely regulates TME immune suppression through activating JAK3/STAT3 signaling to modulate cellular sialylation, a regulatory mechanism not reported for other ccRCC-related lncRNAs. In the present study, we conducted in-depth investigations into the mechanism of *LINC01605* from the perspective of sialylation and demonstrated that *LINC01605* not only promotes ccRCC cell proliferation but also induces exhaustion of tumor-infiltrating CD8^+^ T cells. Our research fills a critical unaddressed gap in the current understanding of *LINC01605*’s functions in TME regulation in ccRCC and establishes a novel framework for predicting clinical outcomes and therapeutic responses in ccRCC patients.

A close relationship has been identified between tumor-infiltrating immune cells and response to immunotherapy as well as prognosis. With the antitumor activity of antigen-specific CD8^+^ T cells as the mechanistic basis, after treatment with ICIs, both the quantity and activity of CD8^+^ T cells have been observed to increase (60). Previous studies show that ccRCC is a highly immune-infiltrated tumor (29, 61). Our risk model revealed a higher abundance of infiltrating CD8^+^ T cells, with fewer B cells and CD4^+^ T cells in the high-risk subgroup than in the low-risk subgroup. Increased infiltration of CD8^+^ memory cytotoxic T cells and Th1 cells into tumors has been consistently linked with favorable clinical outcomes in numerous cancer types (62). However, in ccRCC, a high density of CD8^+^ T cells is correlated with a poor clinical outcome (37). This is consistent with the conclusions derived using our risk model. This is mainly due to the progenitor exhausted population of CD8^+^ T cells, which responds to anti-PD-1 therapy, eventually changes into terminally exhausted cells (63). Studies have confirmed the negative correlation between the exhausted phenotype and prognosis, and higher levels of immune checkpoint molecules were observed in the T-cell-exhausted microenvironment in ccRCC (37, 64). These immune checkpoint proteins are critical regulators of cancer immune escape. Our analysis of *LINC01605* showed that patients with high *LINC01605* expression had more abundant immune checkpoint proteins in CD8^+^ T cells and higher sialic acid levels on the tumor cell membrane, indicating a poor anti-PD-1 immunotherapeutic effect. However, when high levels of CD8^+^ T cells are combined with low *LINC01605* expression, patients had a more favorable prognosis, which indicates that *LINC01605* may mediate the immunosuppressive TME. These results suggest that the genes of the risk model have prognostic significance and can guide the development of individualized therapies through prediction of response to immunotherapy.

As we mentioned before, *LINC01605* may induce an immunosuppressive TME through the increase of sialic acid levels on the cell membranes of tumor cells. Sialoglycans on tumor cells can be involved in tumor cell-cell interactions within the TME, and have been suggested to form a barrier that prevents immune cells from recognizing tumor cells (65). Tumor cells can interact with the Siglec family through aberrant sialoglycan expression to regulate immune cell function in the TME. The TME also appears to stimulate Siglec expression on infiltrating immune cells and enhance aberrant sialylation in tumor cells. Therefore, exploring the inhibitory Siglecs in the TME of ccRCC, including the inhibitory Siglecs on CD8^+^ T cells and associated lncRNAs, is an important direction for future research.

While this study uncovers the role of *LINC01605* in ccRCC progression and immune regulation, it has several limitations. First, the clinical sample size for *in vitro* validation (e.g., PBMC-derived CD8^+^ T cell experiments) is relatively modest, which may restrict the generalizability of findings. Larger multicenter cohorts are needed to confirm *LINC01605*’s prognostic value. Second, mechanistic exploration could be deeper—for instance, the specific domains of *LINC01605* mediating IGF2BP2 binding and the downstream cascades linking sialylation to CD8^+^ T cell exhaustion remain unclear. Third, the PBMC-humanized NCG mouse model lacks a fully functional adaptive immune system, limiting the simulation of clinical ccRCC immune microenvironment.

Future research should focus on three directions: developing *LINC01605*-targeted therapies combined with anti-PD-1/PD-L1 inhibitors to enhance anti-tumor immunity; targeting ST6GALNAC5 or sialic acid metabolism as complementary strategies; and validating *LINC01605* as a predictive biomarker in large-scale clinical trials to stratify patients for personalized therapy. These efforts will accelerate the translation of *LINC01605*-related findings into clinical applications.

## 5 Conclusion

**Figure 7:**
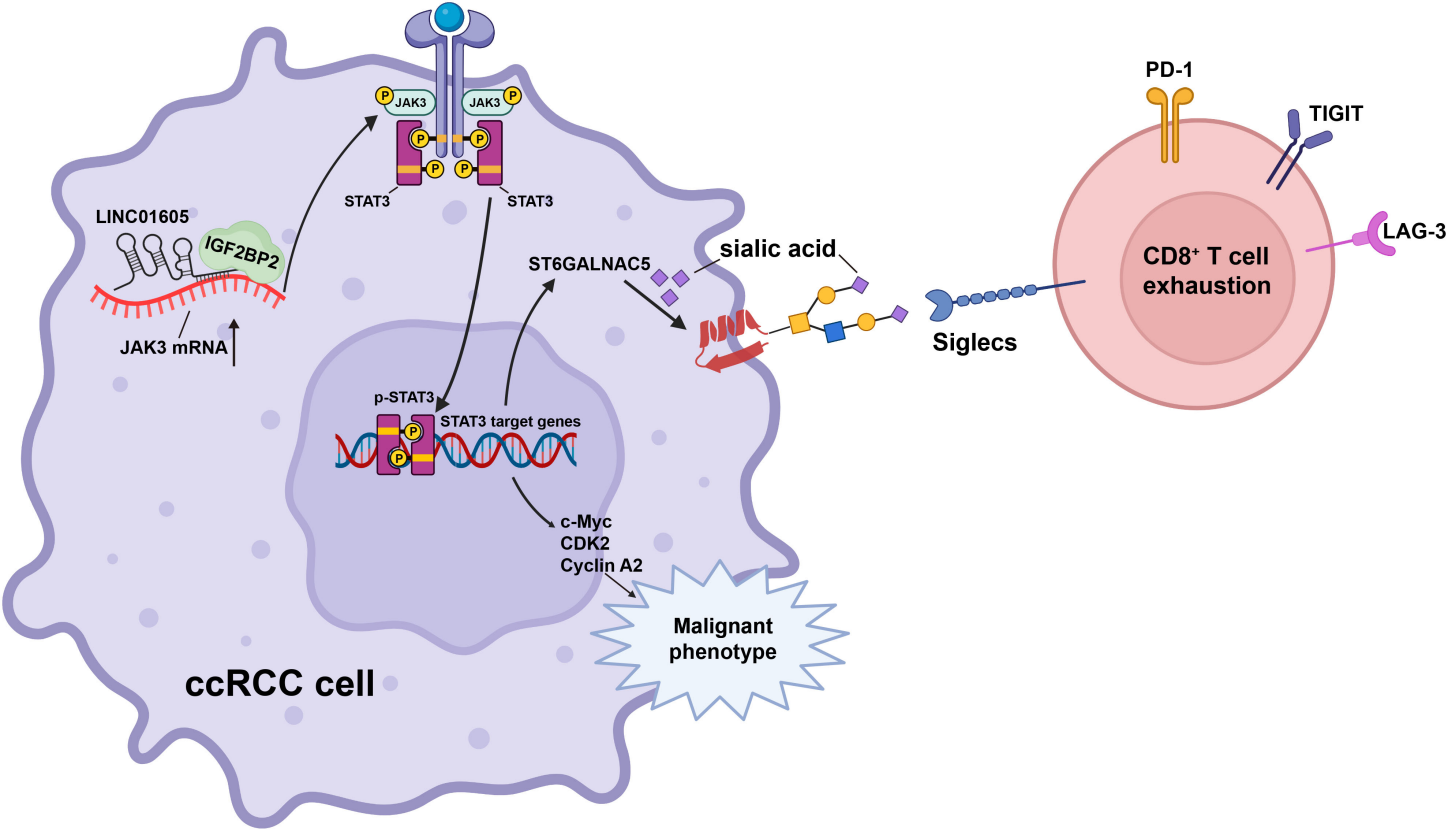
Schematic diagram of the mechanism by which *LINC01605* regulates malignant progression, immune suppression, and cell membrane sialic acid levels in ccRCC cells.

In this study, from the TCGA-KIRC cohort, 12 SIRLs were identified and a risk model was established with high value for prediction of prognosis and response to immunotherapy in ccRCC patients. The hub gene *LINC01605* is involved in tumor progression, CD8^+^ T cell exhaustion, and regulation of sialylation levels in ccRCC. Mechanistically, *LINC01605* upregulates JAK3 expression by recruiting IGF2BP2 to enhance JAK3 mRNA stability, thereby promoting the JAK3/STAT3 pathway (**Figure 7**). Our study contributes to the elucidation of the biological roles of SIRLs in ccRCC tumorigenesis, progression, and TME formation. The identified lncRNAs can be applied to predict ccRCC prognosis as an independent factor, with significant potential to guide the development of effective immunotherapies and targeted therapies for ccRCC.

## Data Availability

The raw data supporting the conclusions of this article will be made available by the authors, without undue reservation.
