# Supplementary material for: Sialylation-immune-related lncRNA LINC01605 promotes tumor-infiltrating CD8+ T cell exhaustion and malignancy of clear cell renal cell carcinoma
Source: Front Immunol. 2026 Jan 16;16:1744278. doi: 10.3389/fimmu.2025.1744278 (PMC12855540; doi:10.3389/fimmu.2025.1744278)
Supplement: Supplementary file 1 [file Table1.docx]

***Supplementary Material***

**Supplementary Figures**

**
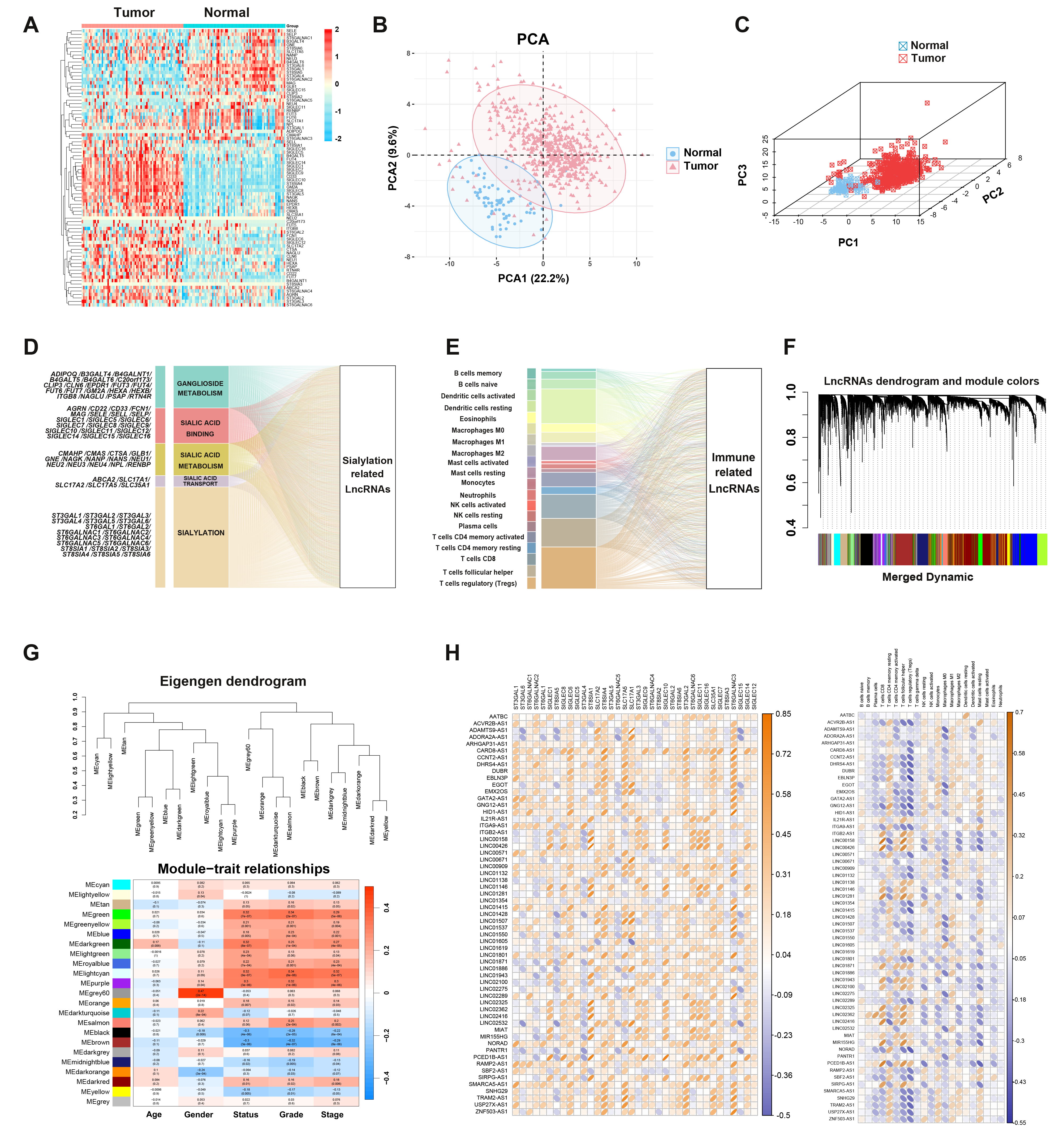
**

**Supplementary Figure 1.** Identification of sialylation-immune-related lncRNAs in ccRCC.

**A.** Heatmap of mRNA expression of 80 genes involved in sialylation in the ccRCC tissues (n = 72) and paired adjacent normal tissues (n = 72) from the TCGA-KIRC cohort.

**B-C.** PCA based on the differentially expressed SRGs in two and three dimensions.

**D.** Sankey diagram depicting sialylation-related lncRNAs and SRGs.

**E.** Sankey diagram illustrating the relationship between immune-correlated lncRNAs and immune fractions.

**F-G.** WGCNA analysis of all lncRNAs in ccRCC from the TCGA-KIRC cohort, and the relationship between the 23 modules and phenotypes.

**H.** The left heatmap reveals the relationship between STs and a proportion of the 66 lncRNAs, and the right heatmap shows the relationship between and immune fractions and a proportion of the 66 lncRNAs. All analyses in Fig.S1 were based on the gene expression data (TPM values) from 528 ccRCC samples and 72 adjacent normal tissue samples in the TCGA-KIRC cohort. n represents the number of samples in **(A)**.

**
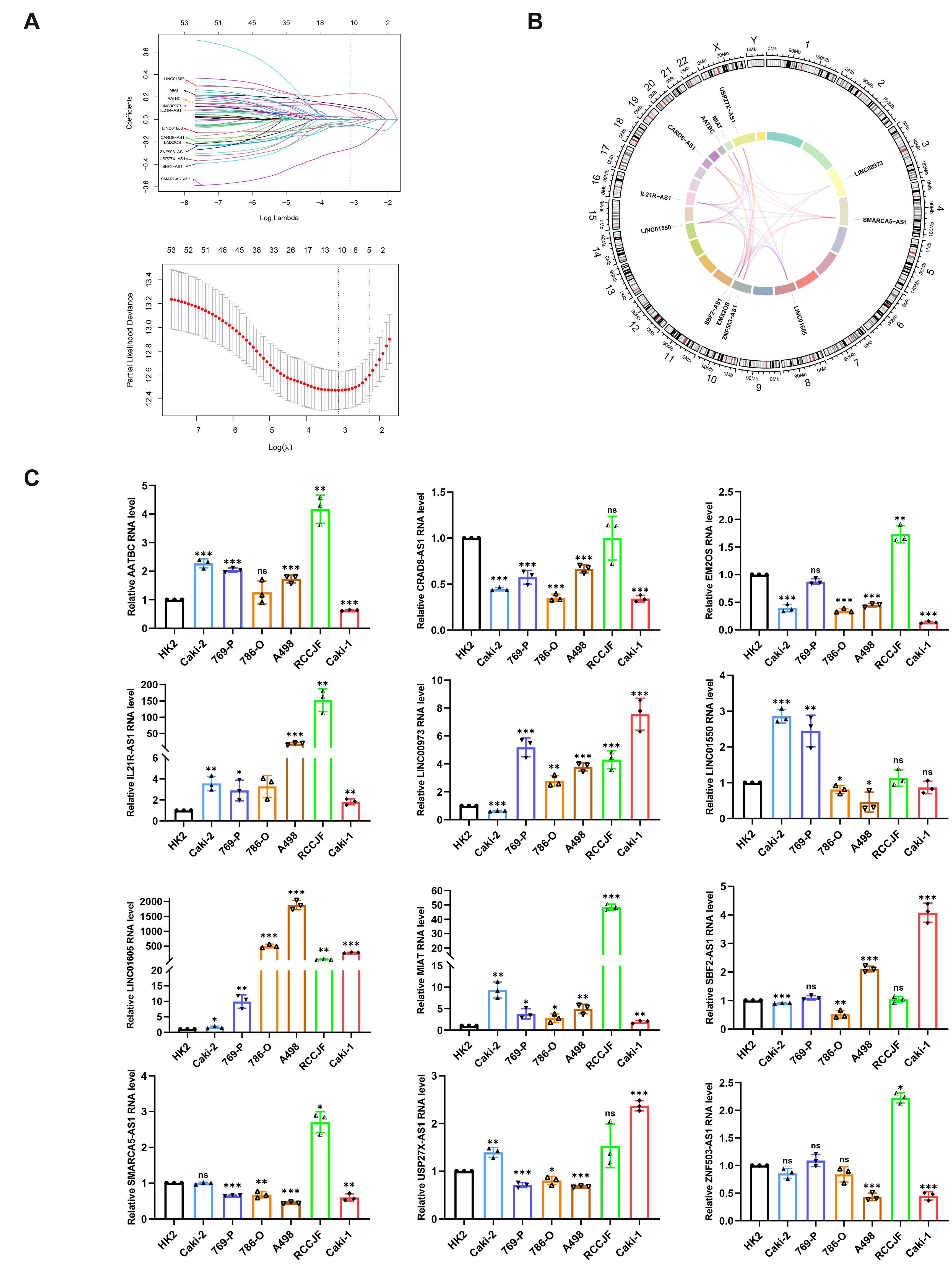
**

**Supplementary Figure 2.** Identification of SIRLs in ccRCC and its validation in ccRCC cell lines.

**A.** A risk model was created using LASSO Cox regression analysis. The optimal parameter (λ) was chosen through the LASSO model, which led to the inclusion of 12 lncRNAs; their corresponding coefficients are displayed.

**B.** The relationships between 12 SIRLs and genomic location shown by chord diagram. A blue link in the diagram indicates a negative correlation, while a red link indicates a positive correlation.

**C.** Relative RNA levels of 12 SIRLs in HK-2 and ccRCC cell lines (n = 3 per group). All bioinformatics analyses in Fig.S2 were based on the gene expression data (TPM values) of 528 ccRCC samples from the TCGA-KIRC cohort. Values are presented as mean ± SD. *P*-values were calculated by one-way ANOVA followed Tukey’s multiple comparisons **(C)**. *p < 0.05; **p < 0.01; ***p < 0.001; ns, not significant. n represents the number of biological replicates in **(C)**.


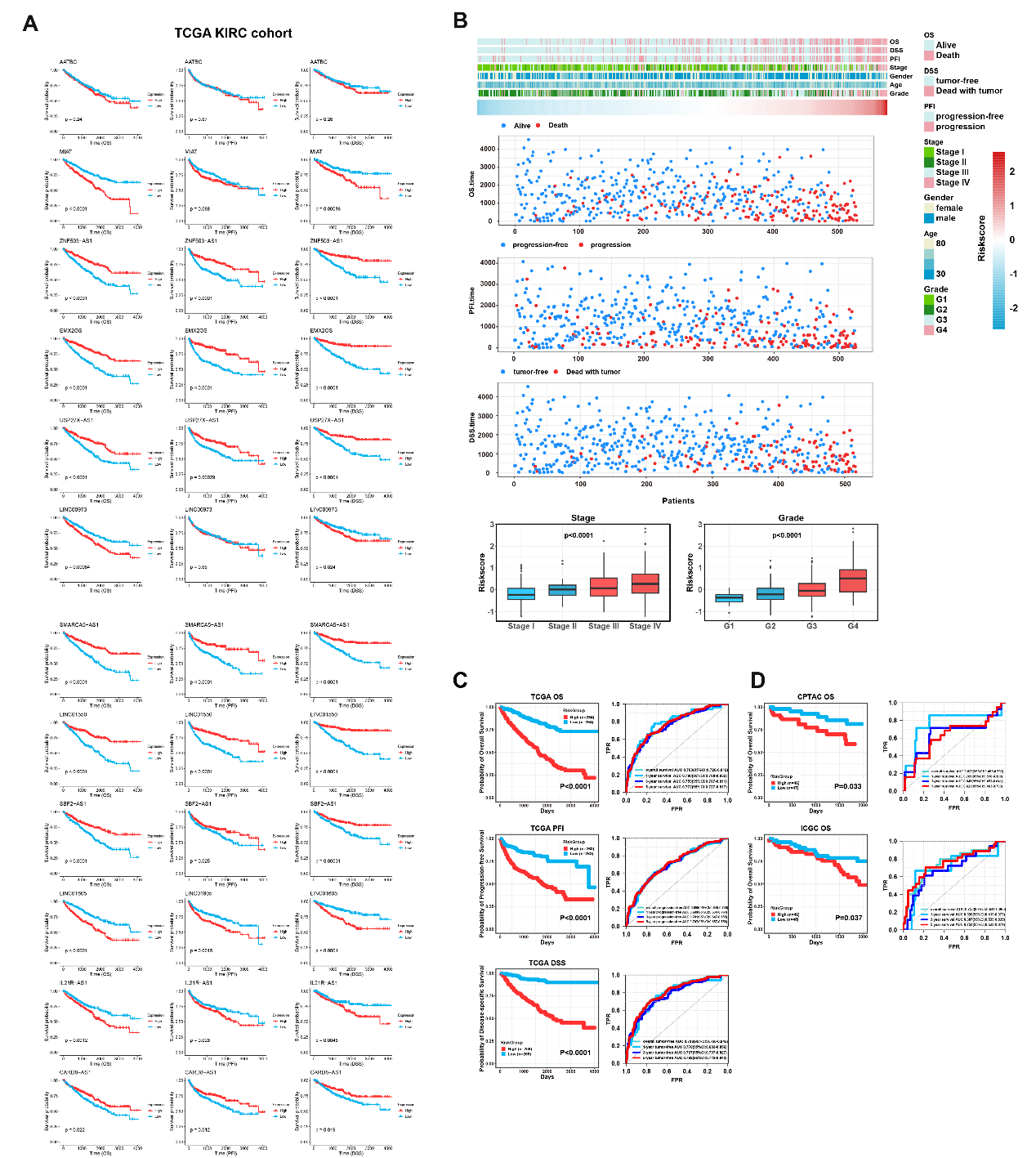


**Supplementary Figure 3.** Development of a risk model for sialylation-immune-related lncRNAs and its association with clinical prognosis in ccRCC.

**A.** Kaplan-Meier plots generated for OS, PFI, and DSS using 12 lncRNAs, using their median expression as the cut-off.

**B.** Scatter plot of correlation between risk score and OS, PFI and DSS, and distribution of clinicopathological characteristics based on SIRL risk score. Box plot depicting the relationships between the risk score and clinicopathological characteristics, including grade and stage.

**C.** In the left panel, the Kaplan-Meier plots compare the OS, PFI, and DSS of the two risk subgroups. The right panel displays the ROC curves for the 1-, 3-, and 5-year prediction of OS, PFI, and DSS using the risk score. All bioinformatics analyses in Fig.S3 were based on the gene expression data (TPM values) and clinical data of 528 ccRCC samples from the TCGA-KIRC cohort.

**D.** The risk model was validated in two independent datasets from CPTAC (n=93) and ICGC (n=91) using Kaplan-Meier plots and ROC curves. Values are presented as mean ± SD. *P*-values were calculated by log-rank (Mantel-Cox) test **(A, C** and **D)**, or one-way ANOVA followed Tukey’s multiple comparisons **(B)**. n represents the number of samples in **(D)**.

**
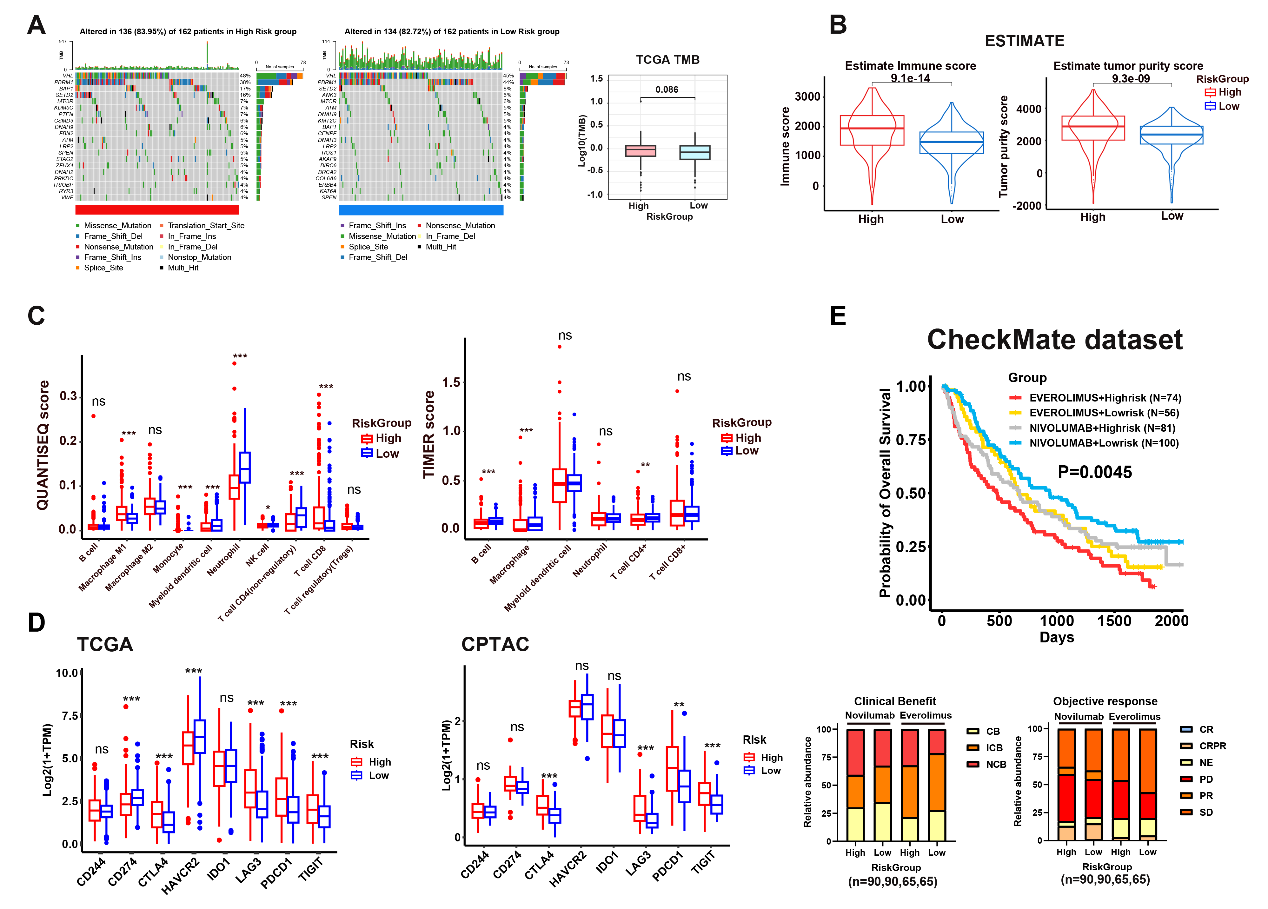
**

**Supplementary Figure 4.** Immune landscapes between high- and low-risk subgroups.

**A.** The oncoplots depict notable disparities in the mutation rates of frequently mutated genes in ccRCC between the two risk subgroups, based on TCGA-KIRC somatic mutation data (n=270). The right panel shows the tumor mutation burden of the two risk subgroups.

**B.** For the two risk subgroups derived from the TCGA-KIRC cohort (n=528), the ESTIMATE algorithm was utilized to assess tumor microenvironment components.

**C.** The QUANTISEQ and TIMER algorithms demonstrated differences in immune cell infiltration levels between the two risk subgroups in the TCGA-KIRC cohort (n=528).

**D.** Expression of CD8^+^ T cell exhaustion markers in the two risk subgroups from the TCGA-KIRC cohort (n=528) and CPTAC cohort (n=103).

**E.** Kaplan-Meier analysis of patients from the CheckMate dataset (n=311). Patients were divided into four groups based on risk score and treatment. The stacked bar charts showed clinical benefit and objective response of the four groups of patients. (“CB” = “clinical benefit”, “ICB” = “intermediate clinical benefit”, “NCB” = “no clinical benefit”, “CR” = “complete response”, “CRPR” = “complete response/partial response”, “NE” = “not evaluated”, “PD” = “progressive disease”, “PR” = “partial response”, “SD” = “stable disease”). Values are presented as mean ± SD. *P*-values were calculated by two-tailed unpaired Student’s t-test **(A-D),** or log-rank (Mantel-Cox) test **(E)**. *p < 0.05; **p < 0.01; ***p < 0.001; ns, not significant. n represents the number of samples in **(A-E)**.


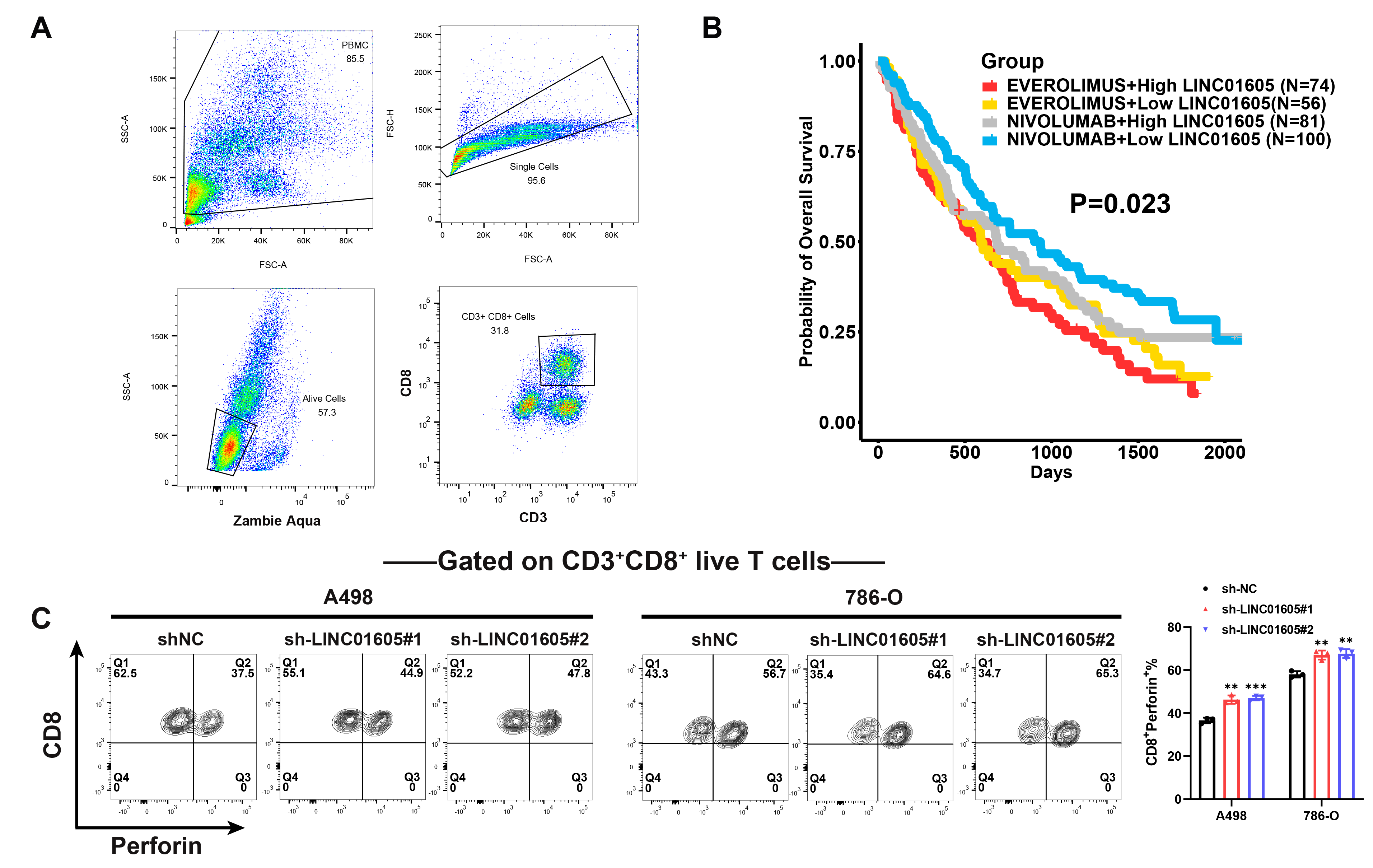


**Supplementary Figure 5.** *LINC01605* mediates the immunosuppressive TME in ccRCC.

**A.** CD3^+^/CD8^+^ cells were identified from PBMCs using flow cytometry.

**B.** Kaplan-Meier analysis of patients from the CheckMate dataset (n=311); patients were divided into four groups based on *LINC01605* expression and treatment.

**C.** Flow cytometry results showed that *LINC01605* knockdown in A498 and 786-O cell lines promoted the secretion of perforin by human CD8^+^ T cells in vitro (n = 3 per group). Values are presented as mean ± SD. *P*-values were calculated by log-rank (Mantel-Cox) test **(B)**, or one-way ANOVA followed Tukey’s multiple comparisons **(C)**. *p < 0.05; **p < 0.01; ***p < 0.001; ns, not significant. n represents the number of samples in **(B)** and the number of biological replicates in **(C)**.


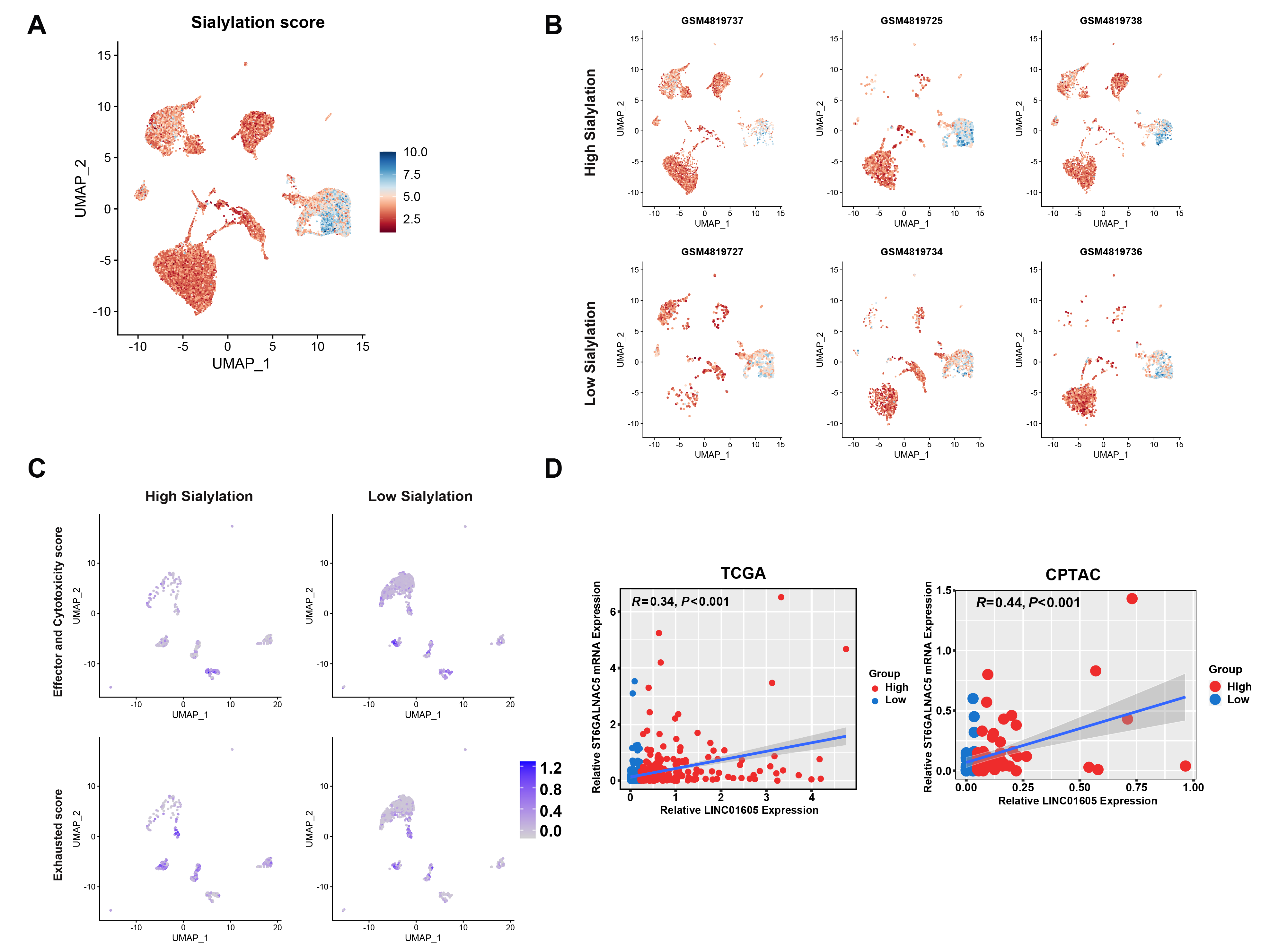


**Supplementary Figure 6.** Sialylation is associated with CD8^+^ T cell exhaustion in ccRCC.

**A-B.** Enrichment pattern of sialylation score in the UMAP plot of six ccRCC samples. Based on the median value of sialylation scores across the six samples, GSM4819737 (sialylation score: -0.02331360), GSM4819725 (sialylation score: -0.02425197), and GSM4819738 (sialylation score: -0.02639415) were assigned to the high-sialylation group, while GSM4819727 (sialylation score: -0.03137073), GSM4819734 (sialylation score: -0.03290425), and GSM4819736 (sialylation score: -0.03416309) were categorized into the low-sialylation group.

**C.** The distribution patterns of exhaustion score, effector score, and cytotoxicity score in the UMAP plot of CD8^+^ T cells between the two sialylation score groups.

**D.** The relationship between *LINC01605* expression and *ST6GALNAC5* mRNA expression based on the TCGA-KIRC cohort (n=528) and the CPTAC cohort (n=103). Pearson’s correlation coefficients (R) and *p*-values were determined via two-tailed unpaired Student’s t-test **(D)**. n represents the number of samples in **(D)**.


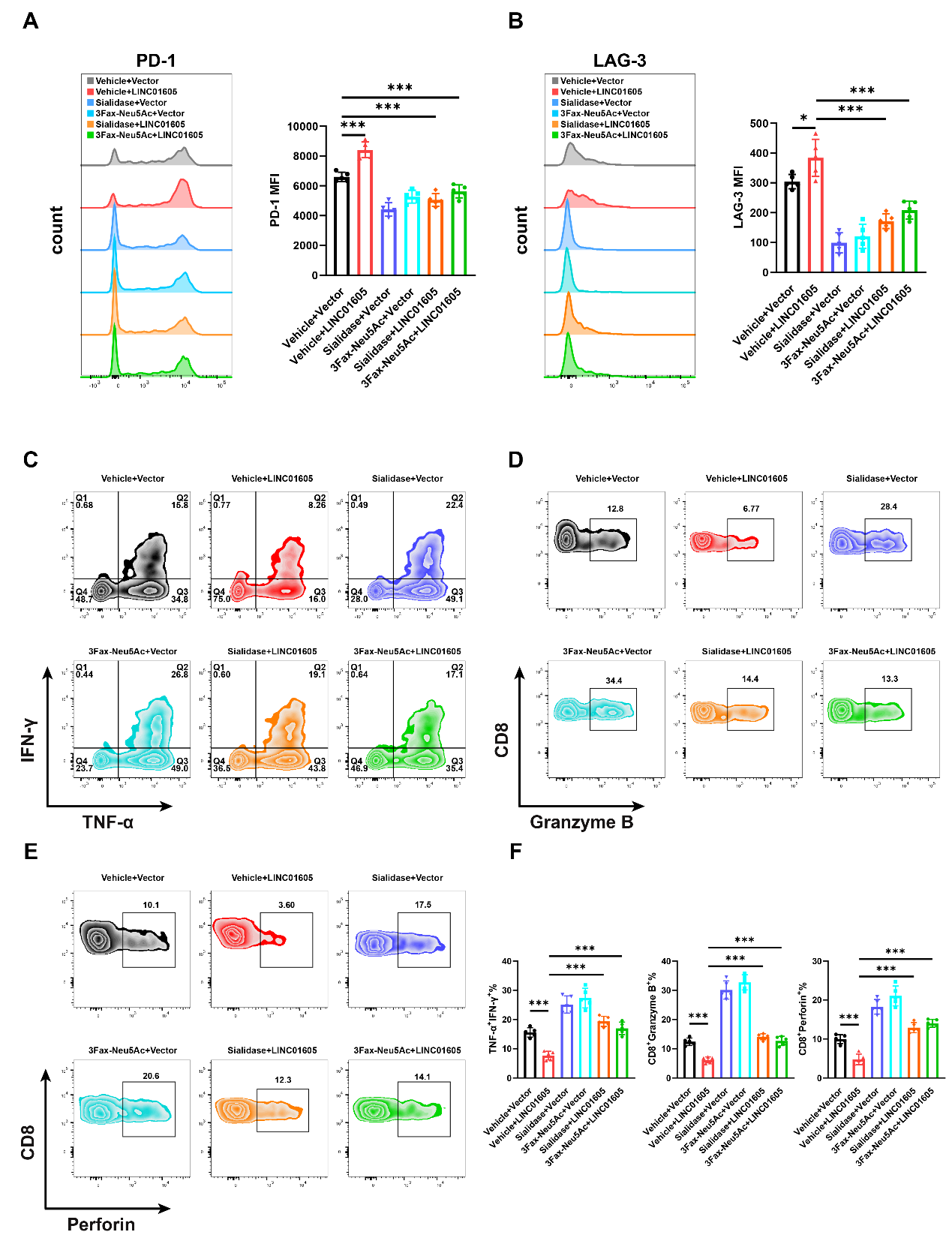


**Supplementary Figure 7.** Sialidase or sialyltransferase inhibitors could rescue the regulatory effect of *LINC01605* on the exhaustion of tumor-infiltrating CD8^+^ T cells and the associated functional impairment.

**A-B.** Flow cytometry results revealed that tumor-infiltrating CD8^+^ T cells from sialidase- or sialyltransferase inhibitor (3Fax-Neu5Ac)-treated, *LINC01605*-overexpressing tumor samples exhibited significantly lower expression of PD-1 and LAG-3 compared with those from *LINC01605*-overexpressing tumor samples without treatment (n = 5 per group).

**C-F.** Flow cytometry results revealed that tumor-infiltrating CD8+ T cells from sialidase- or sialyltransferase inhibitor (3Fax-Neu5Ac)-treated, *LINC01605*-overexpressing tumor samples exhibited significantly increased expression of TNF-α, IFN-γ, GZMB, and perforin compared with those from *LINC01605*-overexpressing tumor samples without treatment (n = 5 per group). Values are presented as mean ± SD. *P*-values were calculated by one-way ANOVA followed Tukey’s multiple comparisons **(A-B**, and **F)**. *p < 0.05; **p < 0.01; ***p < 0.001; ns, not significant. n represents the number of biological replicates in **(A-F)**.


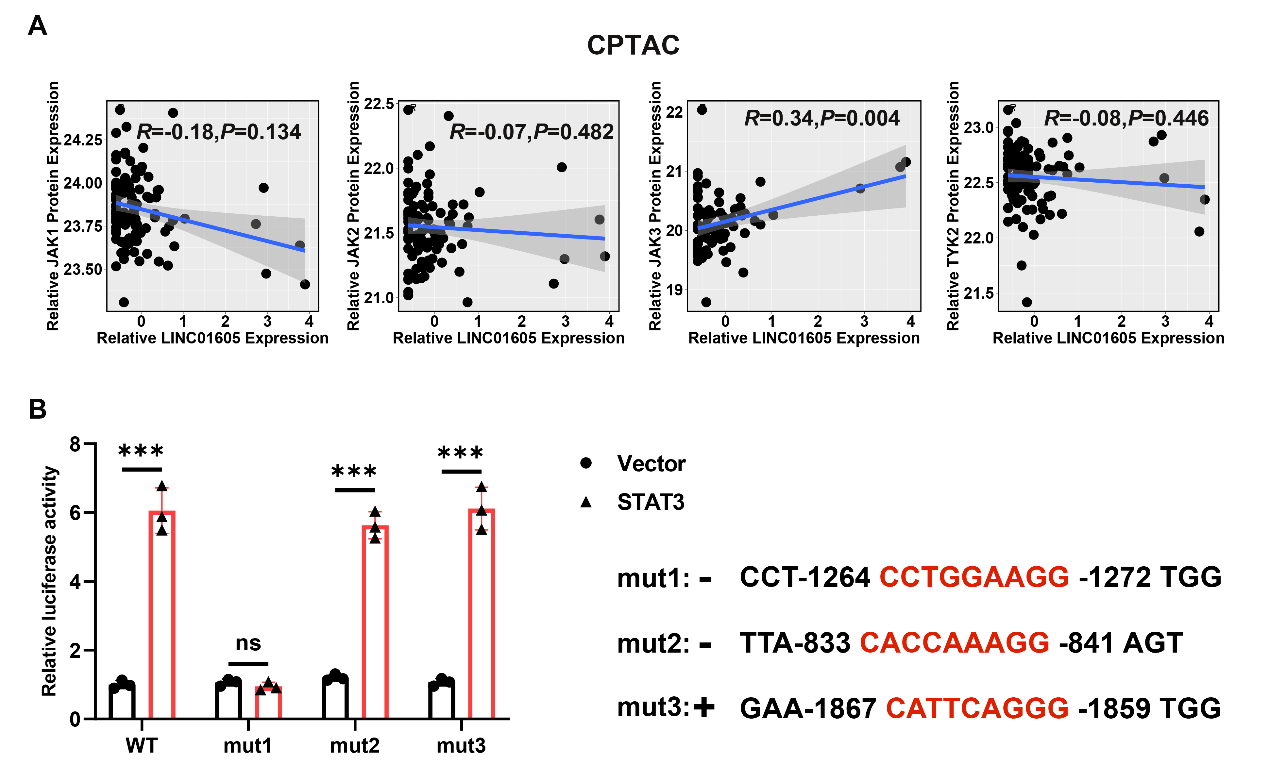


**Supplementary Figure 8.** *LINC01605* is involved in the JAK3/STAT3 signaling pathway, with STAT3 acting as a positive transcriptional regulator of *ST6GALNAC5*.

**A.** Correlation analysis of *LINC01605* expression and protein expression of four JAK molecules based on the CPTAC cohort (n=103).

**B.** Dual-luciferase reporter assays confirmed that mutation 1 (mut1) eliminated STAT3-mediated transactivation of the *ST6GALNAC5* promoter in A498 cells, with red-highlighted bases in the STAT3 binding site indicating mutated sequences (n = 3 per group). Values are presented as mean ± SD. Pearson’s correlation coefficients (R) and *p*-values were determined via two-tailed unpaired Student’s t-test **(A-B)**. *p < 0.05; **p < 0.01; ***p < 0.001; ns, not significant. n represents the number of samples in **(A)** and the number of biological replicates in **(B)**.


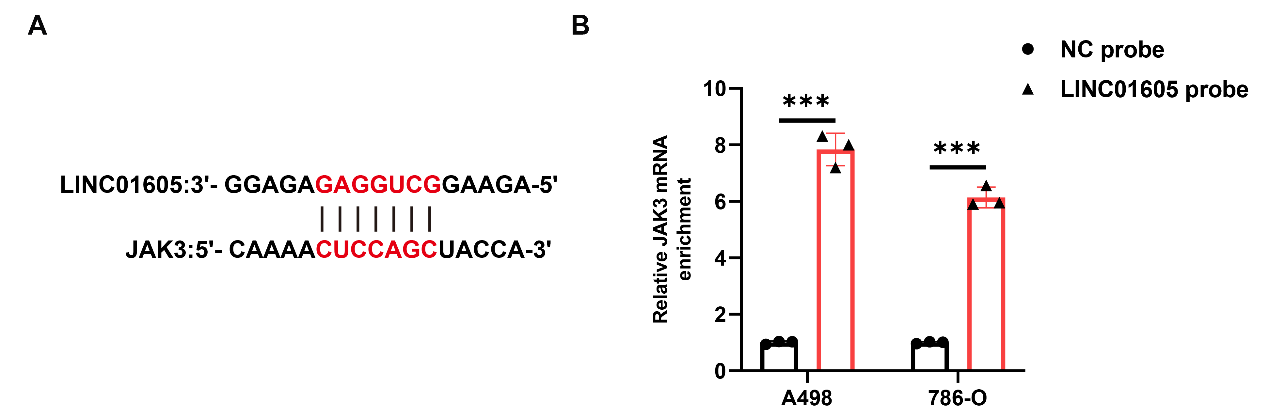


**Supplementary Figure 9.** *LINC01605* binds to JAK3 mRNA in ccRCC cells.

**A.** Sequence BLAST analysis predicted a potential complementary binding region between *LINC01605* and JAK3 mRNA.

**B.** The interaction of *LINC01605* and JAK3 mRNA was further validated using an RNA pull-down assay (n = 3 per group). Values are presented as mean ± SD. *P*-values were calculated by two-tailed unpaired Student’s t-test **(B)**. *p < 0.05; **p < 0.01; ***p < 0.001; ns, not significant. n represents the number of biological replicates in **(B)**.

| **Supplementary Tables**  **Supplementary Table 1. Association between *LINC01605* expression and clinicopathological characteristics of ccRCC patients.**   \| Characteristics \| *LINC01605* expression \| \| *p* value \| \| --- \| --- \| --- \| --- \| \| Low(N=10) \| High(N=10) \| \| Age(years) \|  \|  \| 0.761 \| \| Mean±SD \| 54.9±9.19 \| 56.1±8.14 \|  \| \| Gender \|  \|  \| 0.629 \| \| Female \| 2 (20%) \| 4 (40%) \|  \| \| Male \| 8 (80%) \| 6 (60%) \|  \| \| Smoking status \|  \|  \| 0.650 \| \| Yes \| 3 (30%) \| 5 (50%) \|  \| \| No \| 7 (70%) \| 5 (50%) \|  \| \| Drinking status \|  \|  \| 0.629 \| \| Yes \| 2 (20%) \| 4 (40%) \|  \| \| No \| 8 (80%) \| 6 (60%) \|  \| \| Hypertension \|  \|  \| 0.350 \| \| Yes \| 2 (20%) \| 5 (50%) \|  \| \| No \| 8 (80%) \| 5 (50%) \|  \| \| Diabetes \|  \|  \| 0.629 \| \| Yes \| 2 (20%) \| 4 (40%) \|  \| \| No \| 8 (80%) \| 6 (60%) \|  \| \| Family history \|  \|  \| 0.582 \| \| Yes \| 1 (10%) \| 3 (30%) \|  \| \| No \| 9 (90%) \| 7 (70%) \|  \| \| Tumor size (cm) \|  \|  \| 0.007* \| \| Mean±SD \| 3.37±1.47 \| 5.95±2.25 \|  \| \| WHO/ISUP^a^ grade \|  \|  \| 0.350 \| \| I/II \| 8 (80%) \| 5 (50%) \|  \| \| III/IV \| 2 (20%) \| 5 (50%) \|  \| \| TNM stage \|  \|  \| 0.020* \| \| I/II \| 9 (90%) \| 3 (30%) \|  \| \| III/IV \| 1 (10%) \| 7 (70%) \|  \|   ccRCC clear cell renal cell carcinoma.  ^a^WHO/ISUP World Health Organization/International Society of Urological Pathology grading system.  *p<0.05.  **Supplementary Table 2. Biological reagents and antibodies used in this study** | | |
| --- | --- | --- | --- | --- | --- | --- | --- | --- | --- | --- | --- | --- | --- | --- | --- | --- | --- | --- | --- | --- | --- | --- | --- | --- | --- | --- | --- | --- | --- | --- | --- | --- | --- | --- | --- | --- | --- | --- | --- | --- | --- | --- | --- | --- | --- | --- | --- | --- | --- | --- | --- | --- | --- | --- | --- | --- | --- | --- | --- | --- | --- | --- | --- | --- | --- | --- | --- | --- | --- | --- | --- | --- | --- | --- | --- | --- | --- | --- | --- | --- | --- | --- | --- | --- | --- | --- | --- | --- | --- | --- | --- | --- | --- | --- | --- | --- | --- | --- | --- | --- | --- | --- | --- | --- | --- | --- | --- | --- | --- | --- | --- | --- | --- | --- | --- | --- | --- | --- | --- | --- |
| The following primary antibodies were utilized for western blot analyses | | |
| JAK3 | ab45141 | Abcam |
| p-STAT3 | 39614 | Proteintech |
| STAT3 | 10253-2-AP | Proteintech |
| c-Myc | ab32072 | Abcam |
| CyclinA2 | 18202-1-AP | Proteintech |
| CDK2 | 1022-1-AP | Proteintech |
| PD-L1 | ab205921 | Abcam |
| ST6GALNAC5 | MAB6715-SP | Bio‑Techne |
| IGF2BP2 | 11601-1-AP | Proteintech |
| GAPDH | 60004-1-Ig | Proteintech |
| The following antibodies were used for immunohistochemical (IHC) staining | | |
| PDCD1 | ab52587 | Abcam |
| TIGIT | ab300073 | Abcam |
| LAG3 | 16616-1-AP | Proteintech |
| The following antibodies were used for immunofluorescence (IF) staining | | |
| IGF2BP2 | 11601-1-AP | Proteintech |
| Carbonic anhydrase 9 (CA9) | ET1701-51 | HUABIO |
| The following antibodies were used for flow cytometry analyses | | |
| Zombie Aqua™ Fixable Viability Kit | 423101 | BioLegend |
| Alexa Fluor® 700 anti-human CD45 | 368513 | BioLegend |
| FITC anti-human CD3 | 317305 | BioLegend |
| PerCP/Cyanine5.5 anti-human CD8 | 344709 | BioLegend |
| PE anti-human IFN-γ | 502508 | BioLegend |
| Brilliant Violet 421™ anti-human TNF-α | 502931 | BioLegend |
| APC anti-human/mouse Granzyme B Recombinant | 372203 | BioLegend |
| PE/Dazzle™ 594 anti-human Perforin | 308131 | BioLegend |
| Brilliant Violet 605™ anti-human CD279 (PD-1) | 329923 | BioLegend |
| PE anti-human CD223 (LAG-3) | 369205 | BioLegend |
| FITC-SNA | L32479 | ThermoFisher |

**Supplementary Table 3: Targeted sequences of shRNAs and primers used in this study.**

| **Name** | **Targeted sequences (5’ to 3’)** |
| --- | --- |
| sh-LINC01605#1 | CAAGGTTGTTGTAGGAATTAA |
| sh-LINC01605#2 | CAGGACCAGAGAGAAGACCA |
| sh-IGF2BP2 | AGTGAAGCTGGAAGCGCATAT |
| sh-JAK3 | GCCGAGTTCCTGCGGATGATG |

|  | **sequences (5’ to 3’)** |
| --- | --- |
| LINC01605-F | CAACTCATTCCCGTTACAAACA |
| LINC01605-R | CATCTCAACTGCCTCTGTCTCC |
| SBF2-AS1-F | CACGACCCAGAAGGAGTCTAC |
| SBF2-AS1-R | CCCGGTACCTTCCTGTCATA |
| MIAT-F | GGTCCATGTGGTTAGGGTTG |
| MIAT-R | GGGTTAGTTGGTTGGCAGAA |
| LINC01550-F | AGTGCCCTCAATACGAAG |
| LINC01550-R | AACCTCAGAGCCCACCAC |
| LINC00973-F | TTGAAGGCTTCCTGGTCTGAG |
| LINC00973-R | AGGCTTACATTCCAGCTGTGT |
| IL21R-AS1-F | CTGGTTCTTGTAGCTCCGTG |
| IL21R-AS1-R | CTCACCTTACCCTCATCCTGTG |
| EMX2OS-F | AATGCCACCTCTCTGCTTGACTG |
| EMX2OS-R | AACACCCTTAGACTTCCACACAATCC |
| CARD8-AS1-F | CCTCAGCTGGAATGCCTTCAT |
| CARD8-AS1-R | GGGTTACACACATTCTCGGC |
| AATBC-F | ACCGGGCAAATCTGAAACCA |
| AATBC-R | CGTTGATAACCGGCCTTCCT |
| SMARCA5-AS1-F | GCCATTGTTTCTGCCCACAA |
| SMARCA5-AS1-R | CGGGGTGAAGGAGGCATTTA |
| USP27X-AS1-F | ATCCCACCTCCAGACTGACA |
| USP27X-AS1-R | AGGTGGACCTATGGGCTTCT |
| ZNF503-AS1-F | GTAACTGGTGAAGCCCGGAA |
| ZNF503-AS1-R | GCCCAAACAGCTTCGATTCC |
| JAK3-F | CACCCAAGTCCTGCTGTACA |
| JAK3-R | GTCAAGGATAGCACTGGCCA |
| STAT3-F | ATGGCCCAATGGAATCAGCT |
| STAT3-R | ATCCAAGGGGCCAGAAACTG |
| GAPDH-F | TTCACCACCATGGAGAAGGC |
| GAPDH-R | TGGTTCACACCCATGACGAA |
| ST6GALNAC5-F | ACTGGGCACGGACATTCAAT |
| ST6GALNAC5-R | CTCGGTGTCTGATGCAGTGA |
| **Human ST6GALNAC5 primers for ChIP** |  |
| S1-F | TGCTGCTTTTCCTGCCCTAA |
| S1-R | CAGTGCAACGTGTTTGGGAG |
| S2-F | CCCATGGTTCAGTAGGCCTG |
| S2-R | TGCACTGACGGCGATAGTTT |
| S3-F | CAAGGCTGGCAATGTTGGAC |
| S3-R | TTGGTGCTTTCCGTTGGTCT |
